# Leveraging single‐cell RNA‐seq for uncovering naïve B cells associated with better prognosis of hepatocellular carcinoma

**DOI:** 10.1002/mco2.563

**Published:** 2024-09-09

**Authors:** Qingjia Sun, Rui Gao, Yingxin Lin, Xianchao Zhou, Tao Wang, Jian He

**Affiliations:** ^1^ Department of Otorhinolaryngology Head and Neck Surgery The China‐Japan Union Hospital of Jilin University Changchun China; ^2^ State Key Laboratory of Systems Medicine for Cancer Center for Single‐Cell Omics School of Public Health Shanghai Jiao Tong University School of Medicine Shanghai China; ^3^ School of Mathematics and Statistics The University of Sydney Sydney Australia; ^4^ Univ Lyon, Univ Jean Monnet Saint‐Etienne, INSA Lyon, Univ Lyon 2 Université Claude Roanne France; ^5^ Key Laboratory of Systems Biomedicine Ministry of Education and Collaborative Innovation Center of Systems Biomedicine Shanghai Center for Systems Biomedicine Shanghai Jiao Tong University Shanghai China

**Keywords:** B cell, diethylnitrosamine, hepatocellular carcinoma, prognosis, single‐cell sequencing, transcriptomics

## Abstract

Hepatocellular carcinoma (HCC) is a typical highly heterogeneous solid tumor with high morbidity and mortality worldwide, especially in China; however, the immune microenvironment of HCC has not been clarified so far. Here, we employed single‐cell RNA sequencing (scRNA‐seq) on diethylnitrosamine (DEN)‐induced mouse HCC model to dissect the immune cell dynamics during tumorigenesis. Our findings reveal distinct immune profiles in both precancerous and cancerous lesions, indicating early tumor‐associated immunological alterations. Notably, specific T and B cell subpopulations are preferentially enriched in the HCC tumor microenvironment (TME). Furthermore, we identified a subpopulation of naïve B cells with high CD83 expression, correlating with improved prognosis in human HCC. These signature genes were validated in The Cancer Genome Atlas HCC RNA‐seq dataset. Moreover, cell interaction analysis revealed that subpopulations of B cells in both mouse and human samples are activated and may potentially contribute to oncogenic processes. In summary, our study provides insights into the dynamic immune microenvironment and cellular networks in HCC pathogenesis, with a specific emphasis on naïve B cells. These findings emphasize the significance of targeting TME in HCC patients to prevent HCC pathological progression, which may give a new perspective on the therapeutics for HCC.

## Graphical abstract

The workflow of the study. scRNA‐seq was performed on diethylnitrosamine (DEN)‐induced mouse HCC model to characterize the changes in immune cell composition, lineages, and functional activities in TME during tumorigenesis. Then, the signature genes were mapped for specific cell subpopulations (e.g., naïve B cells) to TCGA database for the HCC RNA‐seq dataset. Finally cell interaction analysis was employed to reveal B cell activation in both mouse and human samples.

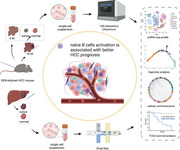

## 1 INTRODUCTION

The compelling studies have indicated that cancer immunotherapies have significantly improved clinical outcomes in some patients with colon, lung, breast, liver cancers, and melanoma.^1^, ^2^, ^3^, ^4^, ^5^ Among common solid tumors, liver cancer has emerged as the third most common cause of cancer‐related mortality worldwide, with hepatocellular carcinoma (HCC) accounting for roughly 90% of the incidence of all liver cancer cases.^6^, ^7^ The clinical success of cancer immunotherapies could mainly depend on in HCC therapies^8^; however, the types, numbers, and activities of the immune cells resided in HCC tumor microenvironment (TME) have not been well defined so far. It has been known that T and B cells are the predominant and extensively studied populations in solid tumor TMEs. Recent advances demonstrated that several factors are associated with clinical responses to immune checkpoint therapies.^9^ However, these results have not been conclusive, and thus the majority of these data have yet to be explored as clinical biomarkers. Previous analyses based on bulk tissue analysis may hide the complexity within different cell types and cell groups, particularly for less abundance of cell types.^10^ Recently, scRNA‐seq provides a potent approach for investigating the cellular heterogeneity in the highly complex TME, by analyzing thousands of single cells,^11^, ^12^, ^13^, ^14^, ^15^ which may ascertain the features of cell type and subtypes, facilitating the prediction and explanation of clinical responses to anticancer agents.

HCC as a type of malignant solid tumor with high morbidity and mortality rates worldwide, particularly in China.^16^ The liver microenvironment has been proven to shape lineage commitment in mosaic mouse models of liver tumorigenesis.^17^ TME of HCC contains a significant level of T cells, as demonstrated by scRNA‐seq; however, the tumor‐infiltrating lymphocytes (TILs) do not kill tumor cells,^14^ which implies that TME is very complex. Therefore, it is crucial to investigate the immune cell composition and status during the development of liver tumors. The liver tissue ecosystem encompasses various cell lineages, including fibroblasts, hepatocytes, epithelial cells, endothelial cells, immune cells, and many others. The process of tumor formation relies on the coordinated interactions and cross‐talk among different cell types and subtypes within the TME ecosystem. Immune cells move from hematopoietic organs to the liver and then create an active immune niche that interactions with stromal cells, which further influences cell differentiation, regeneration, and tumorigenesis. In a specific microenvironment, the major signaling cells determine the composition and characteristics of the cell through the secretion of signaling molecules. Therefore, HCC TME presents a compelling model to characterize various cellular dysregulations and cross‐talk.

The application of scRNA‐seq has provided us with a profound understanding of HCC. Zhang et al., through scRNA‐seq, constructed a cellular atlas of HCC, revealing specific subpopulations of dendritic cells (DCs) and macrophages with immunoregulatory functions, while also identifying early recurrent liver cancer cells employing PD‐L1 for immune evasion.^18^, ^19^ Sharma et al.^20^ found that the HCC tumor microenvironment exhibits fetal‐like reprogramming, with fetal‐like endothelial cells and tumor‐associated macrophages reappearing in the HCC microenvironment. Other studies have indicated the relevance of macrophages to the efficacy of immunotherapy.^21^ Specific subtypes of immune cells, such as neutrophils, also play crucial roles in HCC development and hold promise as novel targets for liver cancer immunotherapy.^22^ In addition to immune cells, stromal cells also play pivotal roles in HCC initiation and progression. For instance, Filliol et al.^23^ discovered that hepatic stellate cells possess dual functions in promoting and inhibiting cancer. Aizarani et al.^24^ identified the procarcinogenic roles of endothelial cells and malignant epithelial cells. These studies underscore the complexity and heterogeneity of HCC, concurrently validating the significant application of single‐cell sequencing in cancer research.

Most studies focus on cross‐sectional at a particular time point, and thus unable to capture the dynamic changes during HCC tumorigenesis. The dissection of the dynamic immune microenvironment during HCC tumorigenesis, including the early and late stages, could provide insight into a profound understanding of cancer development, which could be helpful to the development of targeted immunotherapeutic approaches and aid in the discovery of reliable biomarkers to improve HCC diagnosis and treatment. To address this issue, we introduced animal models. The chemical induction model is one of the most frequently employed models, offering the advantage of mirroring the process of liver cancer formation in animals, similar to that in humans, encompassing injury, fibrosis, and ultimately, liver cancer. Diethylnitrosamine (DEN) is the most frequently utilized hepatocarcinogenic compound, and its induced model serves as a well‐established model for HCC.^25^, ^26^ Studies have demonstrated that intraperitoneal injection of DEN in male mice at a dose of 5 µg/g body weight during the 12–15‐day period resulted in HCC after an average of 44 weeks.^27^ Another study revealed successful induction of HCC in C57BL/6 mice through intraperitoneal administration of DEN (25 mg/kg) for 6 months.^28^ Utilizing the DEN‐induced mouse HCC model, we employed scRNA‐seq to depict the alterations in immune cell composition, lineages, and functional activities within the TME during tumorigenesis.

**FIGURE 1:**
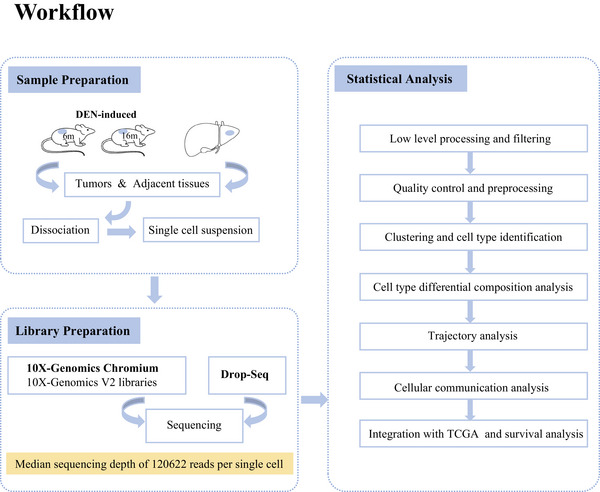
The workflow of the study.

In this study, we designed the experimental workflow (Figure 1).The scRNA‐seq analysis was performed on more than 8000 cells isolated from livers before and after overt HCC development in the DEN‐induced mice. We characterized 36 unique cell subpopulations with distinct gene expression patterns, functional activities, and intercellular interactions. Subsequently, we identified the signature genes of these particular cell subpopulations, and then mapped them to the HCC RNA‐seq dataset of human TCGA database, showing that a population of naïve B cells featured by high CD83 expression was correlated with better prognosis in human HCC. In general, these transcriptomes of immune cells at single‐cell resolution, especially the status transition of T and B cells as well as intercellular interactions between immune cells and hepatocytes, can provide a blueprint of the HCC immune microenvironment and lay a foundation for the understanding of the HCC tumorigenesis and identification of potential biomarkers for HCC diagnosis, therapy, and prognosis.

**FIGURE 2:**
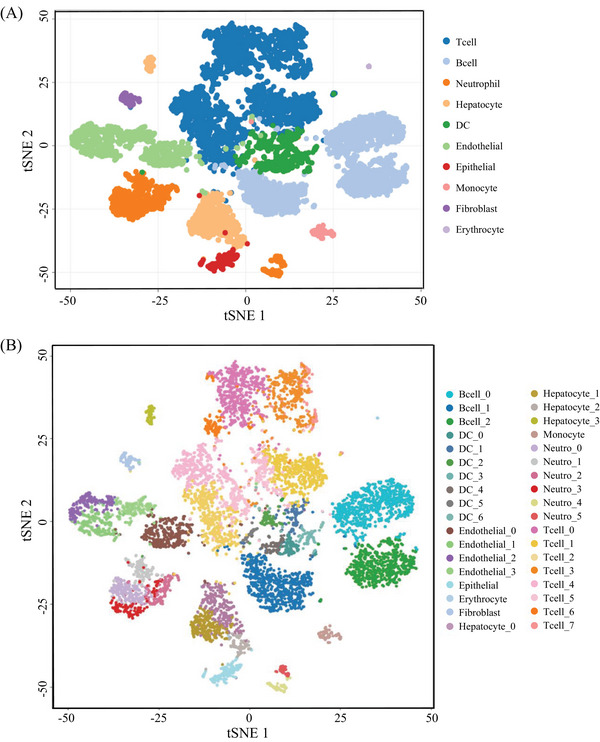
t‐SNE map displaying 8872 cells. (A) Distribution of the 10 cell clusters visualized by t‐distributed stochastic neighbor embedding (t‐SNE), including T cells, neutrophil cells, B cells, hepatocytes, endothelial cells, epithelial cells, dendritic cells (DCs), fibroblasts, and erythrocytes. (B) t‐SNE map of 36 cell subtypes.

## 2 RESULTS

### 2.1 Dynamic landscapes of the cellular composition and characterization during DEN‐induced hepatocellular cancer

**FIGURE 3:**
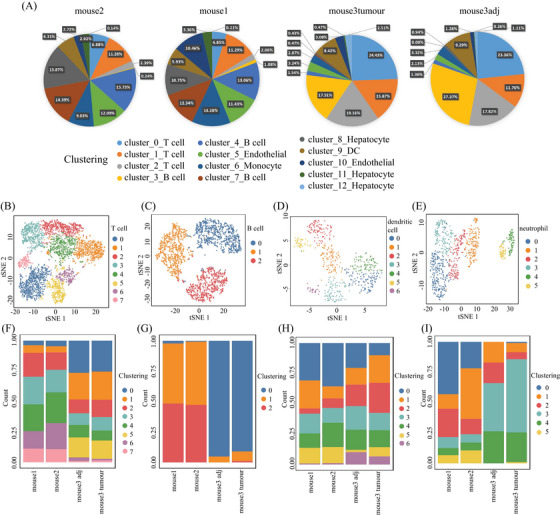
Tissue microenvironment affects the diversity of immune phenotypic states. (A) cell type composition by tissue, (B and F) T cells, (C and G) B cells, (D and H) dendritic cell, and (E and I) neutrophil.

To gain insights into the immune cell landscape during HCC tumorigenesis, we performed scRNA‐seq to profile the transcriptomes of liver cells from DEN‐induced HCC mouse models. We used a total of four tissue samples for this purpose. Two of these samples were whole liver tissues obtained from two individual mice that were 6 months old. The other two samples were obtained from one mouse that was 16 months old, where one sample was a liver tumor and the other was adjacent nontumorous liver tissue. It has been well known that visible liver tumors may be observed in 7−8 months after DEN intraperitoneal injection on 2‐week‐old mice.^25^, ^26^ Thus, in this study, we collected livers from 6 and 16‐month mice after DEN injection, respectively, where the livers of 6‐month‐old mice have no visible tumor but existing inflammatory cells by microscopy, belonging to a precancerous stage, whereas ones from 16‐month‐old mice developed overt tumors with liquefactive necrosis, being in late‐stage (Figure S1). After the quality control, 17,493 genes and 8821 cells were used for downstream analysis (Table S1). Then, we are able to assign these cells into 10 main cell groups, including T cells, neutrophil cells, B cells, hepatocytes, endothelial cells, epithelial cells, DCs, fibroblasts, and erythrocytes, based on these gene markers (Figures 2A and S2), showing that the TME is highly complex with different amount and status of immune cells. In this HCC model, in addition to hepatocytes and erythrocytes (0.2%) that might residual in the livers, there was a large amount of immune cell (78.9%) (Figure 3A) infiltration during HCC tumorigenesis and development.

We then performed the unsupervised clustering on each cell types using the spectral clustering method implemented in Seurat.^29^ Through our refined analysis, we were able to identify a total of 36 subgroups (Figure 2B) with T cells having the largest number of eight subgroups (Figures 3B and F and Table S2), illustrating its diversity characteristics. Here, except for B cells with three subtypes (Figures 3C and G and Table S2), DCs (Figures 3D and H) and neutrophils (Figures 3E and I) were also divided into seven and six subsets, respectively, indicating various responses under different stimulations. Interestingly, the composition of main cell types was similar between tumor and its adjacent (Figure 3A), but the subgroups belonging to DCs and neutrophils were distinctly different (Figures 3H and I and Table S3). T cells were the major immune cell cluster in the HCC TME, with the sample mean of 36.4% (Figure 3A). The mean frequencies of B cells (25.9%), neutrophil (8.6%), hepatocyte (7.9%), DC (6.7%), endothelial (10.1%), epithelial (1.8%), monocyte (1.3%), fibroblast (1.1%), and erythrocyte (0.2%), respectively (Figure 3A), suggesting that, besides the original resident, a large number of immune cells infiltrated in this pathological environment.

Furthermore, we compared the cell type compositions between early and late stages during tumorigenesis (Table S4). In particular, the proportions of T and B cells significantly increased in the late stage (*p* value < 0.001; see *Materials and Methods*) (Figure 3A), whereas the percentage of neutrophils, hepatocytes, endothelial cells, epithelial cells, and fibroblasts decreased during the process, showing cell composition was changing from early to late stages. In the late stage, the total number of T and B cells had exceeded the half of the total number of cells (Figure 3A), where certain subtypes belonging to T and B cells, DCs, and neutrophils were obviously increased in late stages (Figures 3F–I). The collective data, as expected, showed that the immune cell composition and characteristics change during HCC tumorigenesis (between the early and late time points), as indicated by Figure 3, displaying the landscape of the multicellular clusters with different distribution patterns in the HCC microenvironment.

### 2.2 The presence of malignant hepatocytes in the early stage before overt HCC occurrence

**FIGURE 4:**
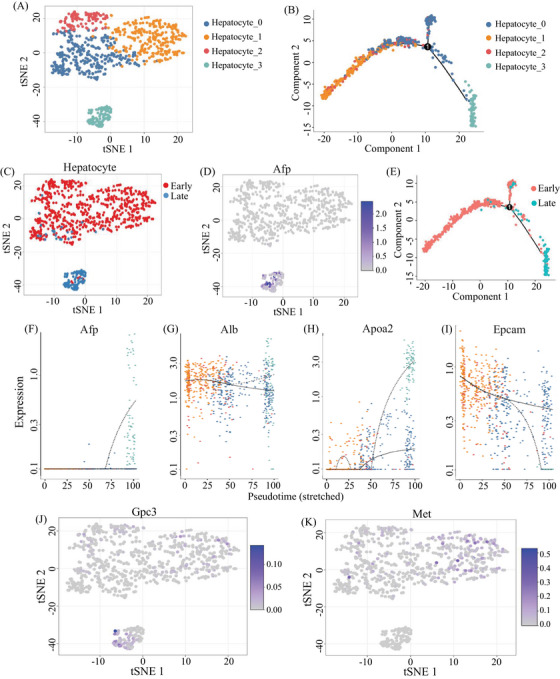
Single‐cell transcriptome reveals malignant show up in precancer. (A) t‐SNE of the subclusters of hepatocyte, (B) pseudo‐trajectory of hepatocytes, (C) early and late stage of hepatocytes, (D) Afp expression patterns in hepatocytes, (E) cell type transition from early to late stage, (F–I) Pseudotime of Afp, Alb, Apoa2, Epcam, (J) Gpc3, and (K) Met expression patterns in hepatocytes.

To investigate the malignant transformation status of hepatocytes during HCC tumorigenesis, we first assigned all hepatocytes from the above livers into four subgroups according to their gene expression patterns (Figure 4A), and then performed the pseudotime trajectory analysis on these cells (Figure 4B), showing that the four subgroups were respectively located at different development or differentiation stages. We further compared the hepatocytes across early and late stages (Figure 4C), showing that the majority of hepatocytes from early and late stages were classified into distinct subgroups. As expected, a group of hepatocytes characterized by Afp‐positive cell subsets is accumulated in late stage, especially in tumors (Figure 4D). Interestingly, however, a few hepatocytes from early stage were clustered in the hepatocyte‐3 subgroup (Figure 4C), which gathered the hepatocytes from tumor of late stage. Moreover, some of these cells also expressed Afp, a well‐known HCC marker (Figure 4D), suggesting that the malignant hepatocytes could exist in the early stage before overt HCC occurrence.

Furthermore, the pseudotime trajectory analysis on hepatocytes from early and late stages showed the continuous process (Figure 4E), suggesting that some of the hepatocytes from the early stage might evolve to tumor formation, along with the switch from Afp‐negative status to Afp‐expressing status during tumorigenesis (Figure 4F). In the switching process, albumin may keep the same level among different subclusters of hepatocyte (Figure 4G), but Apoa2 and Epcam could increase or decrease, respectively (Figures 4H and I). The expression of Afp was dramatically increased in subcluster hepatocyte 3 rather than in other three subclusters. The expression of Apoa2 was higher in subcluster 0 and 3 than in other subclusters. The expression of Epcam was highly expressed in subcluster 3, which is different from the expression patterns of Afp, Alb, and Apoa2. In previous studies based on bulk sequencing, Afp is often negative in the precancerous stage. In this study, we demonstrated that some cells in the early stage could also express Gpc3 at a single cell resolution, along with the liver stem cell marker Met highly expression (Figures 4J and K), where the specific mode of the expression of these stem/progenitor markers might be an indication of early cancer occurrence.

### 2.3 T and B cells exhibit the differential subpopulations between early and late stage during tumorigenesis

**FIGURE 5:**
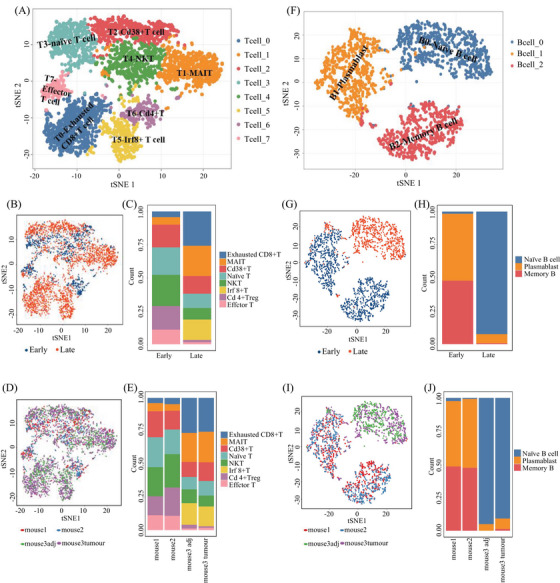
t‐SNE of the subclusters of T cells (A), the t‐SNE of T cells in early and late stage and each mouse (B and D) and the bar plot of T cells in early and late stage (C and E). t‐SNE of the subclusters of B cells (F), the t‐SNE of B cells in early and late stage and each mouse (G and I) and the bar plot of B cells in early and late stage (H and J).

We analyzed the subpopulations and their functional activities of the immune cells, especially T cells with eight subgroups and B cells with three subsets (Figure 5). The results demonstrated the distinct diversity of immune features in precancerous and cancerous lesions. Among eight T‐cell subgroups (Figure 5A, left), except for one reflecting CD4^+^ T cells, seven represented different CD8^+^ T cell subpopulations with known unique signature genes (Figure 5A and Table S2).^30^, ^31^, ^32^ Differential composition analysis revealed that the proportion of T cell subtypes in early and late stages during HCC tumorigenesis was distinctly different (Figure 5). In particular, by fitting a fixed effect generalized linear model (GLM), we found that the proportions of exhausted T cell (T0), MAIT (T1), and Irf8+ T (T5) were significantly increased in the late stage during tumorigenesis (Figures 5C and E), whereas naïve T cell (T3), NKT (T4), and CD4^+^ T (T6) were decreased in late stage (Figures 3A and 5 and Table S3), implying that T cell functions could be suppressed during HCC formation. Three B‐cells subgroups represented naïve B (B0), memory B cells, and plasmablasts, respectively (Figure 5F). There was a clear increase in the proportion of naïve B cell (B0) from the early to late stages during tumorigenesis; by contrast, the proportions of plasmablast (B1) and memory B cell (B2) were dramatically decreased in the late stage (Figure 5H and Table S4), confirmed by the GLM analysis with significant interaction effects between B cell subgroups and stages (*p* < 0.001) (Figures 5H and I). The dramatic change in B cell maturation process suggested TME may inhibit the maturation of naïve B cells during tumorigenesis.

We further validated these findings in a cohort of 365 HCC patients (Table S5). By determining the association between marker genes of naïve B cells (TRAV1‐2, CD161), MAIT cells (SLC4A10, ZBTB16, RORC), and NKT cells (GZMA, GZMK, GZMB, CXCR6) and the survival rate, we discovered that higher expression levels of these genes were correlated with better survival rates. The detailed results can be found in Figure S3. These results doubly confirm our findings at the gene expression level, making our findings more reliable and convincing.

### 2.4 Diverse phenotypes of intratumors PD‐1^+^ T cells

**FIGURE 6:**
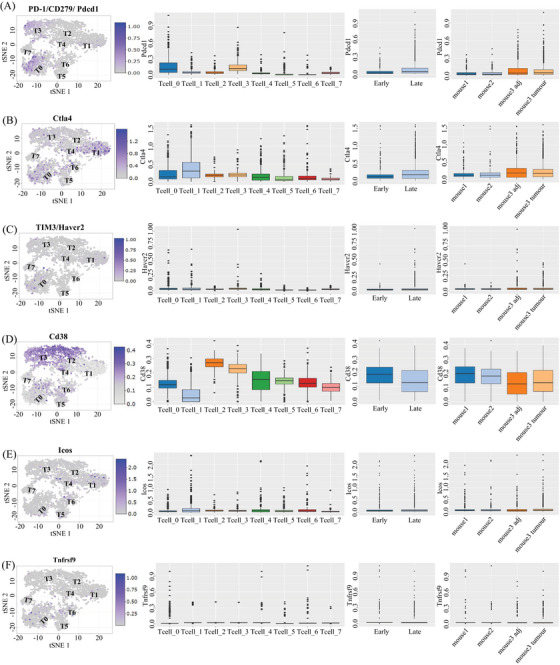
T cell characterized by immunosuppression associated markers (A–F), the expression of these different markers (A‐1 to F‐1), the expression for these markers in early and late stages (A‐2 to F‐2), the expression patterns in tumor and adjacent tissue of these makers.

We observe that T cell populations within tumors are distinguished by different patterns of environmental characteristics. Our study revealed that tumor‐resident T cells may be exposure to different levels of hypoxia, inflammation, and nutrient deprivation. Although many of these responses (e.g., hypoxia or activation) individually represent phenotypic continuums, their combinations may lead to more dissociated states. Figure 6 shows that regulatory T cells (T6) were mostly seen in the CD4^+^ subpopulation and were defined by the coexpression of CD25 and Foxp3. PD‐1^+^ cells were predominantly observed within CD8^+^ subpopulations, particularly in exhausted CD8^+^ T cells (T0) and naive T cells (T3). The T0 subset, characterized by the highest level of PD‐1 expression among CD8^+^ T cells, also exhibited positivity for other coinhibitory receptor Havcr2, activation marker CD38, and costimulatory receptors such as Tnfrsf9 and Icos. This phenotype might be correlated with anti‐PD‐1 treatment response and exhausted T cells.^33^, ^34^ Subpopulations with PD‐1 levels similar were different in the expression on activation markers and costimulatory receptors. Cluster T3 had the same expression pattern as T0 but the activation and costimulatory markers were at a lower level. PD‐1^+^ clusters (T2, T5, and T7) were characterized by a lack of Havcr2 and heterogeneity in the expression of markers such as Tnfrsf9, CD38, and Ctla‐4. Unlike CD4^+^ Regulatory T cells (T6) presented in the early stage, PD‐1^+^ clusters were mainly present in the late stage, but neither of them show a significant difference between tumor and adjacent tissue (Figure 6). All above demonstrated a diversity phenotypic among PD‐1^+^ T cells present in tumorigenesis. Previous findings showed a correlation between the heterogeneity of T cells and the efficacy of anti‐PD‐1 therapies.^35^, ^36^ This provides a clue as to why some immunotherapies are ineffective in the treatment of HCC. We boldly speculate that these different types of PD‐1^+^ T cells may interact with liver cells and other types of immune cells, sustaining an inflammatory environment and promoting disease progression.

### 2.5 Intercellular signaling interaction network could promote HCC development

**FIGURE 7:**
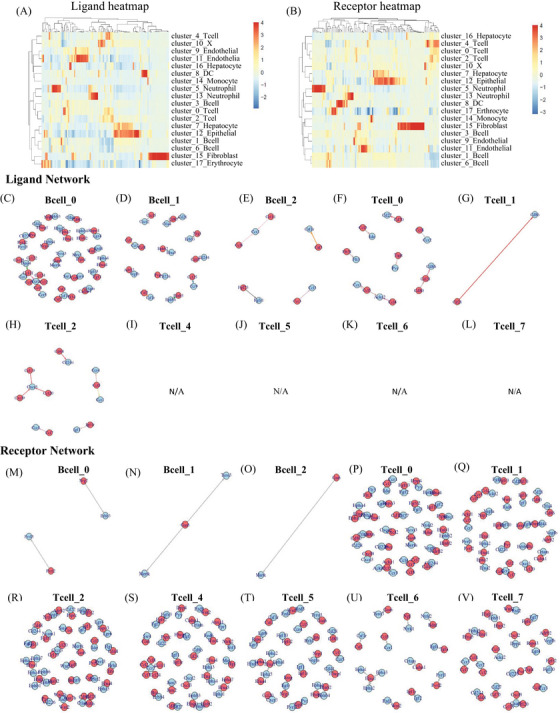
Cell communication predicted by CellPhone DB. Ligand heatmap (A). Receptor heatmap (B). Ligand network of B cells and T cells (C–L). Receptor network of B cells and T cells (M–V).

In order to understand the interactions among different cell types within the HCC microenvironment n DEN‐induced mice, we employed a ligand–receptor interacting repository by the diffusion of secreted molecules (www.CellPhone DB.org).^37^, ^38^ We investigated the expression levels of ligands and receptors in each cell type and determined the ligand–receptor pairs that displayed significant cell‐type specificity (Figure S4). This could predict interactions between cell populations via specific ligand–receptor binding and generate a potential cellular communication network in the HCC microenvironment. B0‐naïve B cells, hepatocytes, all subtypes of the endothelial and DC6 (Ccl17^+^ DC), which are dominant in the late stage, harbored the highest “outgoing” signals. Hepatocyte 3 (late stage Hepatocyte) and dysfunctional T cells harbored the highest “incoming” signals (Table S3). All subtypes of epithelial are harbored both “outgoing” and “incoming” signals. The results demonstrated that B0‐naïve B cells could bind Ccr2 on the dysfunctional T, by releasing cytokine Ccl2, Ccl24, Ccl8, Ccl11, and bind Cxcr3 by releasing cytokine Ccl20. In this way, naïve B cells could regulate T cells. Meanwhile, hepatocyte3 (late stage hepatocyte) could bind Igf1r with B0‐naïve B cells, by releasing cytokine Igf1. Briefly, the hepatocytes in late stage could regulate B0‐naïve B cells. More interesting, naïve B cell binds Ctla4 with all types of T cells except T7‐effect T cell, by releasing CD86. Figures 7 and S5 showed an outline of selected ligand–receptor interactions, indicating that T cells are likely the “terminal,” receiving single from other cells, especially from endothelial, epithelial, and hepatocytes. B cells are more like “originating,” they could release signal to other cells, at the same time, they can “self‐regulation” (Table S3). Altogether, our data support potential regulation mechanisms of different cell types in HCC TME.

### 2.6 Cell interaction analysis reveals B cell activation in both mouse and human samples

**FIGURE 8:**
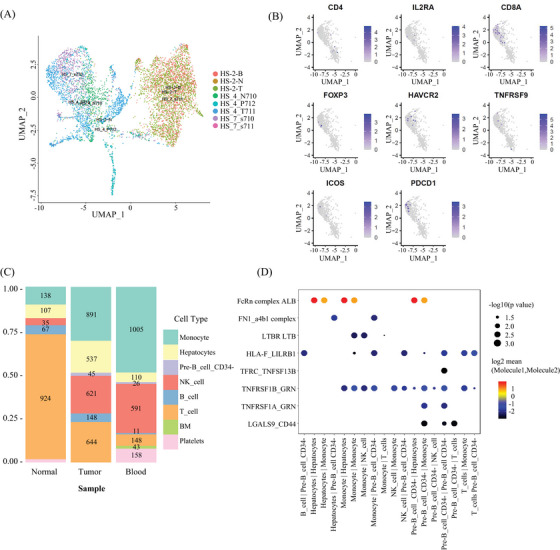
Overview of human single‐cell expression profiles. (A) UMAP of human scRNA‐seq. (B) Eight gene markers labels different types of T cells in tumor. (C) Proportion of cell types in samples from tumor, adjust normal tissue and blood. (D) Cell interactions.

We identified eight basic cell types, including T cells, monocytes, hepatocytes, B cells, CD34‐pre‐B cells, NK cells, BM cells (basement membrane cells), and platelets (only in blood) in three samples from three HCC patients (Figures 8A and B). The clinical information of the three patients is presented in Table S6.) We found that monocytes and NK cells accounted for a greater proportion of tumor cells compared with normal cells (Figure 8C). We tried to build an intercellular signaling interaction network by mapping to the ligand–receptor interacting repository from CellPhone DB (www.CellPhone DB.org).^37^ This time we tried interaction between different cell types and showed the most significant ligand–receptors (Figure 8D). We found high interactions between immune cells, including monocytes and CD34‐pre‐B cells, via molecules such as ALB (ALBINO3‐like protein 1), FcRn complex (IgG receptor FcRn large subunit p51), GRN, and TNFPSF1A.

### 2.7 The markers related to naïve B cells are correlate with better prognosis in HCC patients

**FIGURE 9:**
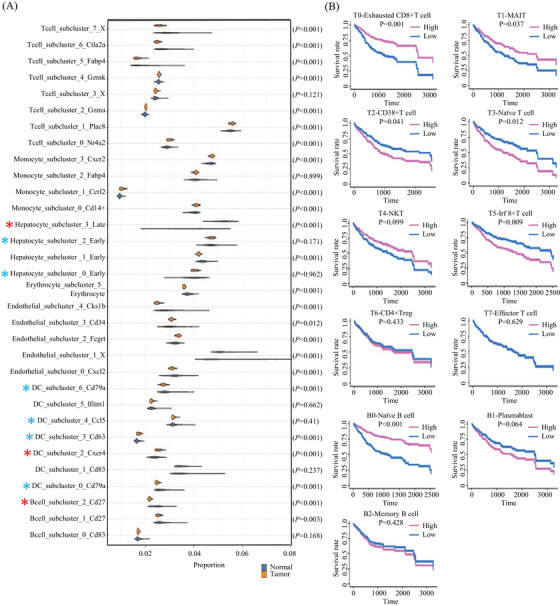
(A) Cell type decomposition with bulk TCGA RNA‐seq data. Red asterisk: bulk data are consistent with scRNA‐seq data; blueline: bulk asterisk is different from scRNA‐Seq data. (B) Correlation between the proportion of cell types with survival rate.

Our finding revealed statistically significant variations in the proportions of multiple cell types between the two sample groups (Figure 9), such as memory B cell (B2), dendritic cell (DC2), and Afp+ hepatocyte (H3). Some clusters share patterns with the bulk sequencing data (Figure 9A) tested by Wilcoxon Rank Sum test. We observed a relevance between the proportion of cell types with survival for immune cells. In particular, we discovered that patients with a higher proportion of MAIT (T1), exhausted CD8^+^ T cell (T0), and naïve B cell (B0) have a better survival rate, while a lower proportion of CD38^+^ T cell (T2), naïve T cell (T3), and Irf8+ T cell (T5) associated with a higher survival rate (Figure 9B). All clues demonstrated the proportion of unmatured B cells could be a key regulator of inflammatory responses in the TME and a potential biomarker of the therapeutics for the HCC.

## 3 DISCUSSION

Advanced cancer, especially liver cancer, is a systemic condition, and our understanding of the immune system's dynamic response at various cancer sites remains incomplete.^18^ ScRNA‐seq offers an alternative approach for exploring the heterogeneity of TME in HCC, by profiling ten thousands of individual cells.^10^, ^11^ The broad‐level analysis of bulk tissue may obscure the intricate variations occurring within individual cells and specific cell clusters, particularly those representing rare cell types.^10^ Additionally, the dynamic alterations in cell composition throughout the progression of HCC tumorigenesis can complicate the differentiation between changes in cellular composition and changes in cellular activity within a specific cell type. Here, we generated transcriptome data by profiling over 8000 individual mouse cells from both tumor and normal tissues, offering a valuable resource for gaining multidimensional insights into the characterization of immune cells in HCC. Among the cells, immune cells make up nearly 70%, with the combined T cell and B cell count exceeding half. Immune cells are vital for us to understand the process of tumor development. This implies that depicting the tumor immune microenvironment is equivalent to understanding the HCC pathogenesis.

T cells as an antitumor response has been widely studied. However, B cell immune response to tumorigenesis and development is poorly defined. Numerous studies have documented a correlation between the presence of tumor‐infiltrating B lymphocytes and favorable clinical outcomes in various cancer types.^39^ The study by Khedr et al.^40^ identified that DEN‐induced markedly significantly higher levels of the signaling growth factors STAT‐3 protein by studying DEN‐intoxicated rats, which strongly suggest that the STAT‐3 signaling pathways are essential for the DEN‐induced hepatocarcinogenesis. Similarly, Liu et al.^41^ also found that HERC2 promoted the malignant phenotype and stemness of HCC cells through the Janus kinase 2 (JAK2)/STAT3 signaling pathway, and then validated this finding using DEN‐induced mouse. It is well known that after antigen recognition and binding to naive B‐cell membrane surface receptors, some B‐cells modulate the expression of CCR7, then some of them differentiate into plasma cells and produce antibodies (IgM). Interestingly, the IL‐6/JAK2/STAT3 pathway, which regulates immune and inflammatory responses by participating in the signaling of a variety of growth factors and cytokines, is activated to promote the differentiation of naïve B cells into plasma cells, leading to the expansion of the inflammatory response and the injury of liver tissue.^42^ All these above demonstrated that a high percentage of Naive B cells might be a key factor in delaying the progression of HCC, prolonging the lifespan of HCC patients, and increasing the survival rate, and tumor‐associated B cells have the potential to serve as valuable biomarkers for early cancer diagnosis and as prognostic indicators for various cancer types.

The gene signatures of B‐cell, especially B‐TILs, have been linked to a positive prognosis in various cancer types, such as breast and ovarian cancers.^43^, ^44^ Schmidt's group demonstrated that the presence of B‐TILs correlates with a positive clinical prognosis, encompassing both breast cancer and NSCLC.^45^, ^46^, ^47^ These investigations have consistently validated the favorable prognostic association of B cells. Here, we used the single‐cell RNA‐seq data to decompose the cell types of the TCGA LIHC bulk RNA‐seq data. We compared the cell type proportion between the primary solid tumor samples and normal solid tissue samples and found that patients with a higher proportion of naïve B cell (B0) have a better survival rate. We also found that the expression level of TRAV1‐2 and CD161, which are predominate markers of naïve B cells, are significant positively associated to better survival rate of HCC patients. Furthermore, immunohistochemical staining of CD20, serving as a surrogate marker for B cells, was also identified as having prognostic significance for B‐TILs in breast cancer.^45^, ^47^ And CD20, a canonical marker of naïve B cell, becomes low expression after naïve B cell matures. So we believe that the results of sequencing analysis of samples from the DEN‐induced HCC mouse model could demonstrate the proportion of naïve B cells could be a key regulator of inflammatory responses in the TME and a potential biomarker of the therapeutics for the HCC.

B cells constitute a diverse population consisting of functionally distinct subsets, which play roles in both promoting and suppressing tumor‐related immune responses. The equilibrium among these subtypes can significantly impact tumor progression and behavior.^45^, ^48^, ^49^ Additional evidence continues to support the notion that B cells possess the capability to identify antigens, govern antigen processing and presentation, and initiate as well as fine‐tune T‐cell and innate immune responses.^45^ In our study, the heterogeneity of B cells and the increase in late‐stage T cells suggesting B cells may regulate the quantity and status of T cells. In addition to their role in immune regulation through antibodies and antibody–antigen complexes, B cells influence the activities of other immune cells through antigen presentation, costimulation, and the secretion of cytokines.^50^, ^51^ All of these pieces of evidence substantiate the pivotal role played by B cells in both cellular and humoral immune responses against cancer, underscoring their significant implications for cancer detection and therapeutic interventions.

In summary, our comprehensive analysis of immune cells at various time points highlights the dynamic nature of immune cell behavior within the TEM. Our investigation has unveiled distinct lineage and migratory patterns among liver cells and immune cells in precancerous, tumor, and normal tissues in cancer‐afflicted mice. To benefit the broader research community, we have developed an interactive web‐based tool accessible at the following links (http://shiny.maths.usyd.edu.au/Yingxin/HCC10xData/; https://yingxinlin.shinyapps.io/HCC10xDataShiny/), which allows users to analyze and visualize single‐cell data for specific genes of interest. Our dataset represents a valuable resource for further exploration, offering deeper insights into liver cancer biology and potential novel targets and biomarkers for therapeutic advancements.

## 4 MATERIALS AND METHODS

### 4.1 DEN‐induced HCC mouse model

We utilized a DEN‐induced HCC mouse model, employing C57BL/6 mice procured from the Shanghai Laboratory Animal Center. Our study included three DEN‐induced mice, consisting of two 6‐month‐old mice and one 16‐month‐old mouse, all pathologically diagnosed with HCC. To induce HCC, we administered an intraperitoneal injection of 25 mg/kg DEN on the twelfth day after birth. Subsequently, we harvested whole liver tissues from the 6‐month‐old DEN‐induced mice and paired fresh tumors along with adjacent liver tissues from the 16‐month‐old DEN‐induced mouse.

### 4.2 Tissue dissociation and single cell suspension preparation

Fresh tissue samples were initially cut into approximately 1 mm^3^ pieces. Subsequently, they were gently homogenized by repeatedly pressing through a 70 µm cell strainer (BD) into a solution of phosphate‐buffered saline (PBS) (Invitrogen) containing 10% fetal bovine serum. The residual material on the 70 µm cell strainer was collected and subjected to a 20‐min digestion process with 0.05% collagenase IV on a shaker at 37°C. The resulting mixture was then filtered through a 40 µm cell strainer into the previously obtained cell suspension. Following this, the suspended cells were centrifuged at 200×*g* for 5 min. After discarding the supernatant, the pelleted cells were exposed to erythrocyte lysis buffer (Solarbio) and incubated on ice for 5 min to remove red blood cells. The cell pellets were subsequently resuspended in a buffer solution after being washed twice with 1 × PBS.

### 4.3 Hematoxylin and eosin staining

Slides were prepared by heating them on a slide warming table for 5−10 min, with the treatment being repeated if necessary. Subsequently, the tissue sections were hydrated through a graded alcohol series, with each concentration held in decreasing order for 1−2 min (starting from 100% alcohol and decreasing to 50%). The slides were then washed in tap water.

For staining, the sections were immersed in hematoxylin for 3−5 min and rinsed under running tap water for a brief period, not exceeding 5 min. Differentiation was achieved by placing the slides in 1% acid alcohol (1% HCl in 70% alcohol) for 5 min. (Microscopic examination confirmed the nuclei to be dark purple or reddish‐blue, while the tissues appeared pale.) Following differentiation, slides were rinsed in running tap water until sections reverted to a blue hue, achieved by immersion in an alkaline solution followed by another tap water rinse.

Eosin staining was performed for 30 s, followed by a tap water rinse lasting 1−5 min. To complete the process, dehydration was carried out through a series of increasing alcohol concentrations, and clarity was achieved by immersion in xylene. The stained slides were then observed under a microscope.

### 4.4 Library preparation

Detailed procedure refer to the User Guide of 10× Genomics V2 libraries preparation available at: W020190620596403691451.pdf (ion.ac.cn).

Subsequently, the libraries were subjected to sequencing on an Illumina HiSeq 2000 platform (paired‐end configuration; read 1: 26 cycles; i7 index: 8 cycles; i5 index: 0 cycles; read 2: 98 cycles). The Drop‐Seq method was executed in accordance with the McCarroll Lab's protocol, and you can find comprehensive operational instructions in Drop‐seq—McCarroll Lab.

### 4.5 Low level processing and filtering

For the critical processing steps, Cell Ranger software was employed, including demultiplexing, alignment, barcode counting, and unique molecular identifier (UMI) counting. To ensure data quality, cells with fewer than 100 UMIs were excluded. Subsequently, Seurat was applied for data normalization, dimensionality reduction, unsupervised clustering, and visualization of single‐cell transcriptomes.

### 4.6 Quality control and preprocessing

We employed the k‐means clustering technique to categorize cells based on their total expression and mitochondrial proportion. The integration of multisample data and batch effect correction was accomplished using the Harmony software. Moreover, to ensure the accuracy of downstream analyses, we employed the DoubletFinder software to identify and eliminate potential doublets. Following quality control, we utilized 17,493 genes and 8821 cells for downstream analysis.

### 4.7 Clustering and cell type identification

To delineate and characterize cell types in our dataset, we initiated a clustering analysis using Seurat with the first 20 principal components (PCs). Subsequently, we conducted differential expression analysis employing MAST and differential variability analysis using the Bartlett test to pinpoint marker genes specific to each cluster.

### 4.8 Cell type differential composition analysis

We used the scdney R package (https://github.com/SydneyBioX/scdney) to examine the relationship between cell type proportions and mouse phenotypes. Subsequently, we applied a fixed‐effects GLM to the number of cells within each cell type.

### 4.9 Trajectory analysis

To explore the trajectory of hepatocyte cell development during tumorigenesis and its progression, we employed the Monocle2 software.

### 4.10 Cellular communication analysis

We conducted an analysis inspired by Vento‐Tormo et al.^37^, utilizing the ligand‐receptor pairs available in the CellPhone DB database. To assess cell‐cell interactions, we employed permutation tests on the product of the average gene expression levels of ligands in one cell type and receptors in another.

### 4.11 Integration with TCGA data and survival analysis

We leveraged single‐cell RNA‐seq data to deconvolute cell types within TCGA LIHC bulk RNA‐seq datasets using dtangle. Subsequently, we applied a Cox proportional hazards model to evaluate the association between cell type proportions and survival outcomes.

### 4.12 Statistical analysis

Statistical analyses were conducted using R version 4.2.0. The first 20 PCs of the datasets were clustered using Seurat. Differential expression analysis was subsequently performed employing MAST, and differential variability analysis was conducted using Bartlett's test to identify marker genes specific to each cluster.

## AUTHOR CONTRIBUTIONS

J. H. contributed to the idea, conception, and study design. J. H. and Y. L. collected and analyzed the datasets. J. H., Q. S., and R. G. wrote the manuscript and generated the figures. R. G., T. W., and X. Z. revised and proofread the article. All authors have read and agreed to the published version of the manuscript.

## CONFLICT OF INTEREST STATEMENT

The authors declare that there are no conflict of interest.

## ETHICS STATEMENT

This study was approved by the Ethics Committee of China‐Japan Union Hospital of Jilin University. The approval number for animal experiments is A‐2023‐009, the approval number for clinical experiments is 2023111605. Written informed consent was obtained from all participants.

## Supporting information

Supporting Information

## Data Availability

To access this information, please visit the project's page directly at the following website: https://db.cngb.org/search/project/CNP0004178/ (the specific project number: CNP0004178) and the data are available on request from the authors.

## References

[1] Luoma AM , Suo S , Williams HL , et al. Molecular pathways of colon inflammation induced by cancer immunotherapy. Cell. 2020;182(3):655‐671. e22. 32603654 10.1016/j.cell.2020.06.001 PMC7415717

[2] He J , Lin F , Yang X , Wang D , Tan X , Zhang S . Sustainable synthesis of 2‐arylbenzoxazoles over a cobalt‐based nanocomposite catalyst. Org Process Res Dev. 2016;20(6):1093‐1096.

[3] Kieffer Y , Hocine HR , Gentric G , et al. Single‐cell analysis reveals fibroblast clusters linked to immunotherapy resistance in cancer. Cancer Discov. 2020;10(9):1330‐1351. 32434947 10.1158/2159-8290.CD-19-1384

[4] Zheng H , Peng X , Yang S , et al. Targeting tumor‐associated macrophages in hepatocellular carcinoma: biology, strategy, and immunotherapy. Cell Death Discov. 2023;9(1):65. 36792608 10.1038/s41420-023-01356-7 PMC9931715

[5] Kurt FGO , Lasser S , Arkhypov I , Utikal J , Umansky V . Enhancing immunotherapy response in melanoma: myeloid‐derived suppressor cells as a therapeutic target. J Clin Invest. 2023;133(13):e170762. 37395271 10.1172/JCI170762 PMC10313369

[6] Rumgay H , Arnold M , Ferlay J , et al. Global burden of primary liver cancer in 2020 and predictions to 2040. J Hepatol. 2022;77(6):1598‐1606. 36208844 10.1016/j.jhep.2022.08.021 PMC9670241

[7] He J , Meng M , Wang H . A novel prognostic biomarker LPAR6 in hepatocellular carcinoma via associating with immune infiltrates. J Clin Transl Hepatol. 2022;10(1):90‐103. 35233377 10.14218/JCTH.2021.00047 PMC8845155

[8] Prieto J , Melero I , Sangro B . Immunological landscape and immunotherapy of hepatocellular carcinoma. Nat Rev Gastroenterol Hepatol. 2015;12(12):681‐700. 26484443 10.1038/nrgastro.2015.173

[9] Topalian SL , Taube JM , Anders RA , Pardoll DM . Mechanism‐driven biomarkers to guide immune checkpoint blockade in cancer therapy. Nat Rev Cancer. 2016;16(5):275‐287. 27079802 10.1038/nrc.2016.36 PMC5381938

[10] De Strooper B , Karran E . The cellular phase of Alzheimer's disease. Cell. 2016;164(4):603‐615. 26871627 10.1016/j.cell.2015.12.056

[11] Habib N , Avraham‐Davidi I , Basu A , et al. Massively parallel single‐nucleus RNA‐seq with DroNc‐seq. Nat Methods. 2017;14(10):955‐958. 28846088 10.1038/nmeth.4407 PMC5623139

[12] He J , Meng M , Zhou X , Gao R , Wang H . Isolation of single cells from human hepatoblastoma tissues for whole‐exome sequencing. STAR Protoc. 2023;4(1):102052. 36853859 10.1016/j.xpro.2023.102052 PMC9876968

[13] Tirosh I , Izar B , Prakadan SM , et al. Dissecting the multicellular ecosystem of metastatic melanoma by single‐cell RNA‐seq. Science. 2016;352(6282):189‐196. 27124452 10.1126/science.aad0501 PMC4944528

[14] Liu Y , Xun Z , Ma K , et al. Identification of a tumour immune barrier in the HCC microenvironment that determines the efficacy of immunotherapy. J Hepatol. 2023;78(4):770‐782. 36708811 10.1016/j.jhep.2023.01.011

[15] He J , Lin YX , Meng M , Li JQ , Yang JYH , Wang H . Construction of a human cell landscape of COVID‐19 infection at single‐cell level. Aging Dis. 2021;12(3):705‐709. 34094635 10.14336/AD.2021.0301 PMC8139199

[16] Zhou X , Meng M , Wu Y , et al. Protocol to dissociate and isolate wide‐diversity single cells by density gradient centrifugation from human hepatoblastoma tissue. STAR Protoc. 2023;4(3):102449. 37459235 10.1016/j.xpro.2023.102449 PMC10511933

[17] Seehawer M , Heinzmann F , D'artista L , et al. Necroptosis microenvironment directs lineage commitment in liver cancer. Nature. 2018;562(7725):69‐75. 30209397 10.1038/s41586-018-0519-y PMC8111790

[18] Zhang Q , He Y , Luo N , et al. Landscape and dynamics of single immune cells in hepatocellular carcinoma. Cell. 2019;179(4):829‐845. e20. 31675496 10.1016/j.cell.2019.10.003

[19] Sun Y , Wu L , Zhong Y , et al. Single‐cell landscape of the ecosystem in early‐relapse hepatocellular carcinoma. Cell. 2021;184(2):404‐421. e16. 33357445 10.1016/j.cell.2020.11.041

[20] Sharma A , Seow JJW , Dutertre CA , et al. Onco‐fetal reprogramming of endothelial cells drives immunosuppressive macrophages in hepatocellular carcinoma. Cell. 2020;183(2):377‐394. e21. 32976798 10.1016/j.cell.2020.08.040

[21] Yu J , Green MD , Li S , et al. Liver metastasis restrains immunotherapy efficacy via macrophage‐mediated T cell elimination. Nat Med. 2021;27(1):152‐164. 33398162 10.1038/s41591-020-1131-x PMC8095049

[22] Xue R , Zhang Q , Cao Q , et al. Liver tumour immune microenvironment subtypes and neutrophil heterogeneity. Nature. 2022;612(7938):141‐147. 36352227 10.1038/s41586-022-05400-x

[23] Filliol A , Saito Y , Nair A , et al. Opposing roles of hepatic stellate cell subpopulations in hepatocarcinogenesis. Nature. 2022;610(7931):356‐365. 36198802 10.1038/s41586-022-05289-6 PMC9949942

[24] Aizarani N , Saviano A , Sagar , et al. A human liver cell atlas reveals heterogeneity and epithelial progenitors. Nature. 2019;572(7768):199‐204. 31292543 10.1038/s41586-019-1373-2 PMC6687507

[25] Zhang W , Zhangyuan G , Wang F , et al. The zinc finger protein Miz1 suppresses liver tumorigenesis by restricting hepatocyte‐driven macrophage activation and inflammation. Immunity. 2021;54(6):1168‐1185. e8. 34038747 10.1016/j.immuni.2021.04.027

[26] Li Z , Zhou Y , Jia K , et al. JMJD4‐demethylated RIG‐I prevents hepatic steatosis and carcinogenesis. J Hematol Oncol. 2022;15(1):1‐19. 36333807 10.1186/s13045-022-01381-6 PMC9636772

[27] Vesselinovitch S , Koka M , Mihailovich N , Rao K . Carcinogenicity of diethylnitrosamine in newborn, infant, and adult mice. J Cancer Res Clin Oncol. 1984;108(1):60‐65. 6746718 10.1007/BF00390974 PMC12253112

[28] Cheng J , Zhong Y , Chen S , et al. Gab2 mediates hepatocellular carcinogenesis by integrating multiple signaling pathways. FASEB J. 2017;31(12):5530. 28842424 10.1096/fj.201700120RR PMC5690380

[29] Butler A , Hoffman P , Smibert P , Papalexi E , Satija R . Integrating single‐cell transcriptomic data across different conditions, technologies, and species. Nat Biotechnol. 2018;36(5):411‐420. 29608179 10.1038/nbt.4096 PMC6700744

[30] Chen L , Flies DB . Molecular mechanisms of T cell co‐stimulation and co‐inhibition. Nat Rev Immunol. 2013;13(4):227‐242. 23470321 10.1038/nri3405 PMC3786574

[31] Kurioka A , Walker LJ , Klenerman P , Willberg CB . MAIT cells: new guardians of the liver. Clin Transl Immunol. 2016;5(8):e98. 10.1038/cti.2016.51 PMC5007630 27588203

[32] Böttcher JP , Beyer M , Meissner F , et al. Functional classification of memory CD8+ T cells by CX3CR1 expression. Nat Commun. 2015;6(1):8306. 26404698 10.1038/ncomms9306 PMC4667439

[33] Ahmadzadeh M , Johnson LA , Heemskerk B , et al. Tumor antigen–specific CD8 T cells infiltrating the tumor express high levels of PD‐1 and are functionally impaired. Blood. 2009;114(8):1537‐1544. 19423728 10.1182/blood-2008-12-195792 PMC2927090

[34] Daud AI , Loo K , Pauli ML , et al. Tumor immune profiling predicts response to anti–PD‐1 therapy in human melanoma. J Clin Invest. 2016;126(9):3447‐3452. 27525433 10.1172/JCI87324 PMC5004965

[35] Li J , Wu C , Hu H , et al. Remodeling of the immune and stromal cell compartment by PD‐1 blockade in mismatch repair‐deficient colorectal cancer. Cancer Cell. 2023;41(6):1152‐1169. 37172580 10.1016/j.ccell.2023.04.011

[36] Wang X , Zha H , Wu W , et al. CD200+ cytotoxic T lymphocytes in the tumor microenvironment are crucial for efficacious anti–PD‐1/PD‐L1 therapy. Sci Transl Med. 2023;15(679):eabn5029. 36652534 10.1126/scitranslmed.abn5029

[37] Vento‐Tormo R , Efremova M , Botting RA , et al. Single‐cell reconstruction of the early maternal–fetal interface in humans. Nature. 2018;563(7731):347‐353. 30429548 10.1038/s41586-018-0698-6 PMC7612850

[38] Förster R , Davalos‐Misslitz AC , Rot A . CCR7 and its ligands: balancing immunity and tolerance. Nat Rev Immunol. 2008;8(5):362‐371. 18379575 10.1038/nri2297

[39] Wang S‐S , Liu W , Ly D , Xu H , Qu L , Zhang L . Tumor‐infiltrating B cells: their role and application in anti‐tumor immunity in lung cancer. Cell Mol Immunol. 2019;16(1):6‐18. 29628498 10.1038/s41423-018-0027-x PMC6318290

[40] Khedr OMS , El‐Sonbaty SM , Moawed FSM , Kandil EI , Abdel‐Maksoud BE . Lactobacillus acidophilus ATCC 4356 exopolysaccharides suppresses mediators of inflammation through the inhibition of TLR2/STAT‐3/P38‐MAPK pathway in DEN‐induced hepatocarcinogenesis in rats. Nutr Cancer. 2022;74(3):1037‐1047. 34085875 10.1080/01635581.2021.1934490

[41] Liu YZ , Xu QS , Deng F , et al. HERC2 promotes inflammation‐driven cancer stemness and immune evasion in hepatocellular carcinoma by activating STAT3 pathway. J Exp Clin Cancer Res. 2023;42(1):38. 36721234 10.1186/s13046-023-02609-0 PMC9890722

[42] Mirlekar B , Wang Y , Li SR , et al. Balance between immunoregulatory B cells and plasma cells drives pancreatic tumor immunity. Cell Rep Med. 2022;3(9):100744. 36099917 10.1016/j.xcrm.2022.100744 PMC9512696

[43] Iglesia MD , Vincent BG , Parker JS , et al. Prognostic B‐cell signatures using mRNA‐seq in patients with subtype‐specific breast and ovarian cancer. Clin Cancer Res. 2014;20(14):3818‐3829. 24916698 10.1158/1078-0432.CCR-13-3368 PMC4102637

[44] Lundberg A , Li B , Li R . B cell‐related gene signature and cancer immunotherapy response. Br J Cancer. 2022;126(6):899‐906. 34921229 10.1038/s41416-021-01674-6 PMC8927337

[45] Tsou P , Katayama H , Ostrin EJ , Hanash SM . The emerging role of B cells in tumor immunity. Cancer Res. 2016;76(19):5597‐5601. 27634765 10.1158/0008-5472.CAN-16-0431

[46] Lohr M , Edlund K , Botling J , et al. The prognostic relevance of tumour‐infiltrating plasma cells and immunoglobulin kappa C indicates an important role of the humoral immune response in non‐small cell lung cancer. Cancer Lett. 2013;333(2):222‐228. 23370224 10.1016/j.canlet.2013.01.036

[47] Mahmoud S , Lee A , Paish E , Macmillan R , Ellis I , Green A . The prognostic significance of B lymphocytes in invasive carcinoma of the breast. Breast Cancer Res Treat. 2012;132:545‐553. 21671016 10.1007/s10549-011-1620-1

[48] Wei Y , Huang C‐X , Xiao X , et al. B cell heterogeneity, plasticity, and functional diversity in cancer microenvironments. Oncogene. 2021;40(29):4737‐4745. 34188249 10.1038/s41388-021-01918-y

[49] Bai L , Chen W , Chen J , et al. Heterogeneity of Toll‐like receptor 9 signaling in B cell malignancies and its potential therapeutic application. J Transl Med. 2017;15(1):1‐10. 28241765 10.1186/s12967-017-1152-5 PMC5329966

[50] Martin F , Chan AC . B cell immunobiology in disease: evolving concepts from the clinic. Annu Rev Immunol. 2006;24:467‐496. 16551256 10.1146/annurev.immunol.24.021605.090517

[51] Stoycheva D , Simsek H , Weber W , Hauser AE , Klotzsch E . External cues to drive B cell function towards immunotherapy. Acta Biomater. 2021;133:222‐230. 33636402 10.1016/j.actbio.2021.02.026

