# Supplementary material for: Leveraging single‐cell RNA‐seq for uncovering naïve B cells associated with better prognosis of hepatocellular carcinoma
Source: MedComm (2020). 2024 Sep 9;5(9):e563. doi: 10.1002/mco2.563 (PMC11381656; doi:10.1002/mco2.563)
Supplement: Supplementary file 1 — Supporting Information [file MCO2-5-e563-s001.pdf]

Leveraging Single-Cell RNA-Seq for Uncovering Naïve B Cells Associated with Better Prognosis of Hepatocellular Carcinoma

Qingjia Sun[1#], Rui Gao[2#], Yingxin Lin[3#], Xianchao Zhou[2], Tao Wang[4], Jian He [2,5*]

1 Department of Otorhinolaryngology Head and Neck Surgery, The China-Japan Union Hospital of Jilin University, Changchun, China

2 State Key Laboratory of Systems Medicine for Cancer, Center for Single-Cell Omics, School of Public Health, Shanghai Jiao Tong University School of Medicine, Shanghai 200025, China

3 School of Mathematics and Statistics, The University of Sydney, Sydney, Australia

4 Univ Lyon, Univ Jean Monnet Saint-Etienne, INSA Lyon, Univ Lyon 2, Université Claude, Bernard Lyon 1, DISP-UR4570, 42300 Roanne, France

5 Key Laboratory of Systems Biomedicine, Ministry of Education and Collaborative Innovation Center of Systems Biomedicine, Shanghai Center for Systems Biomedicine, Shanghai Jiao Tong University, Shanghai, China

[#] Equal Contribution

*Correspondence to: Jian He, Email: jih003@sjtu.edu.cn.

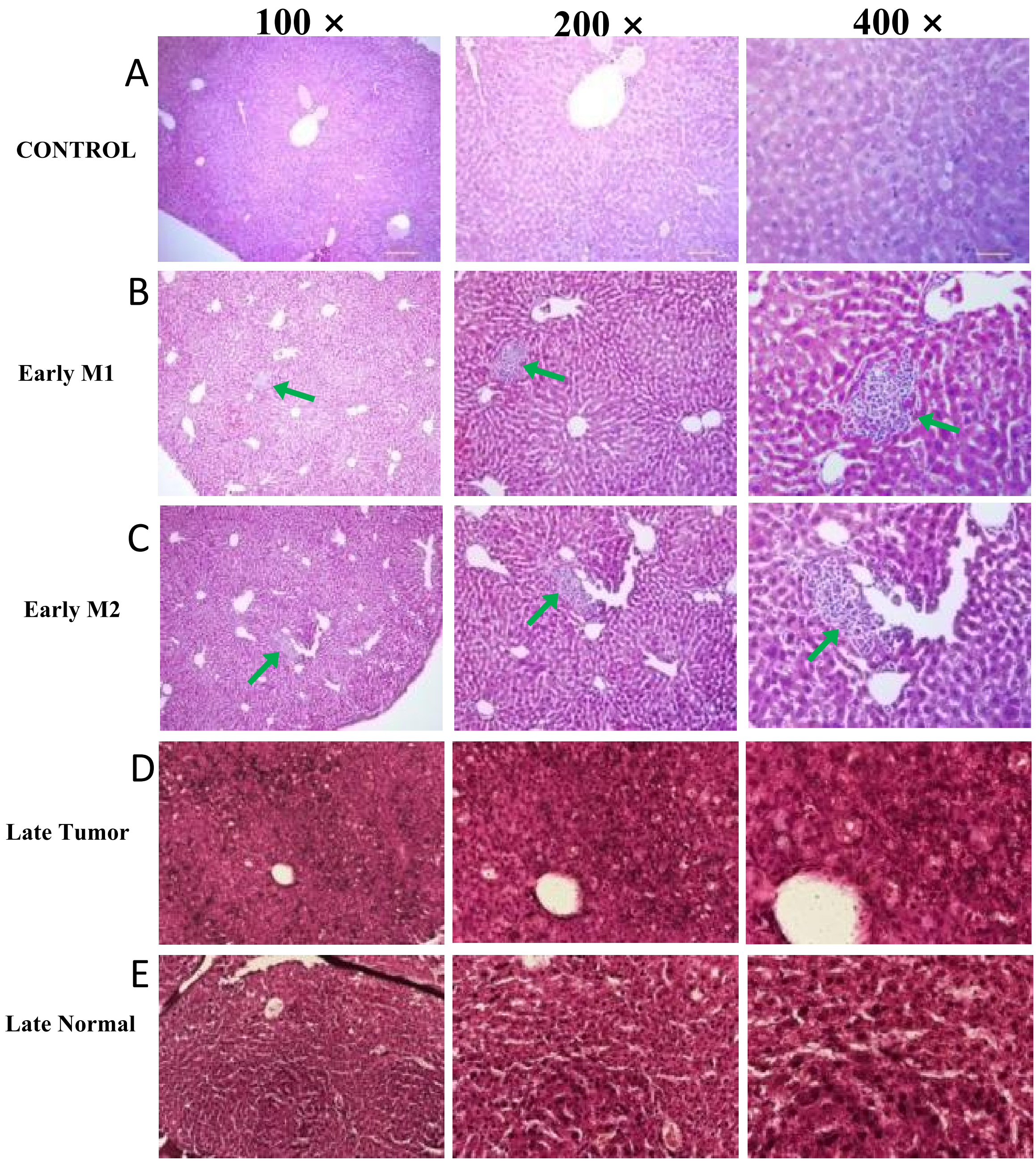

SI-Fig. 1. HE staining of the DEN-induced mice. Early stage (6M after DEN injection) belongs to pre-cancerous stage (second row), have no visible tumor but existing inflammatory cells under microscope (B and C). The late stage is a 16-month-old mice developed over tumors with liquefactive necrosis, we got tumor tissue and normal tissue from the late stage mice (D and E).

SI-FIGURE 2

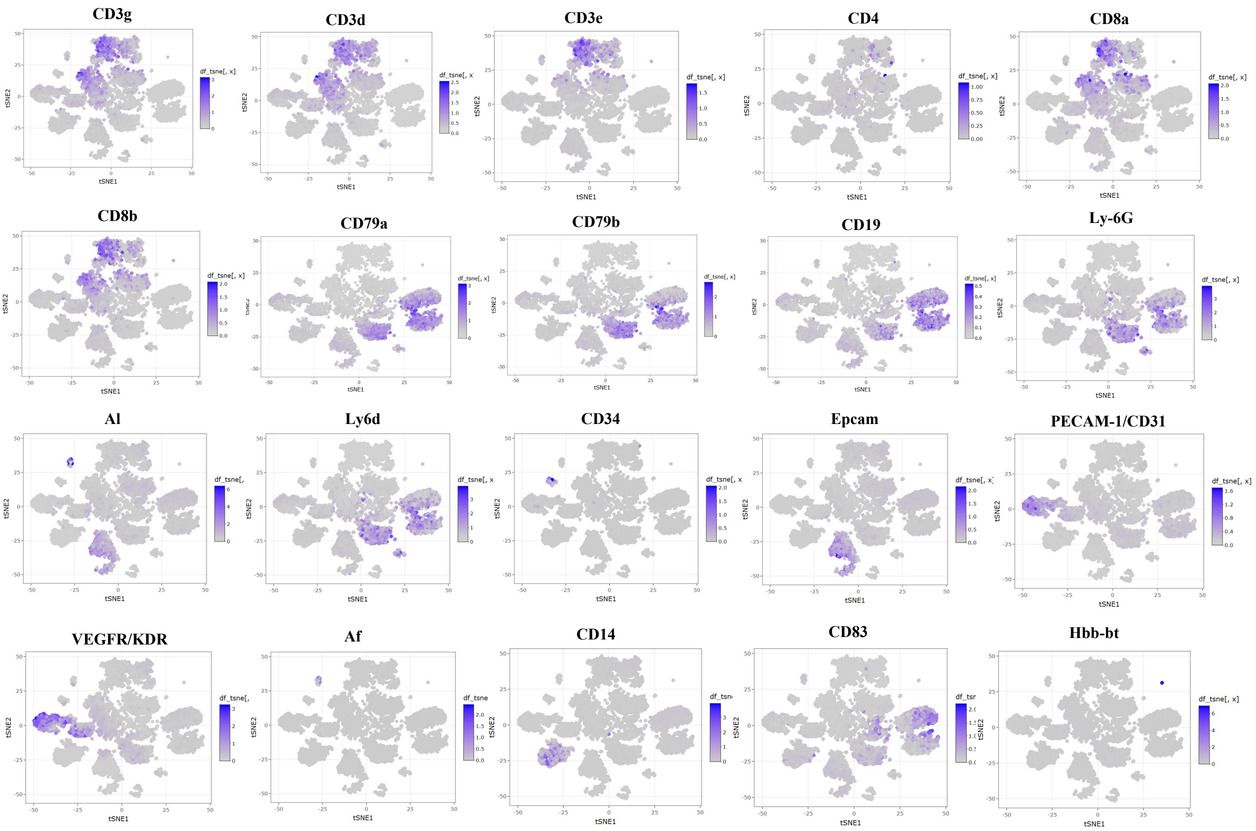

SI-Fig2. Gene markers of each cell types.

SI-FIGURE 3

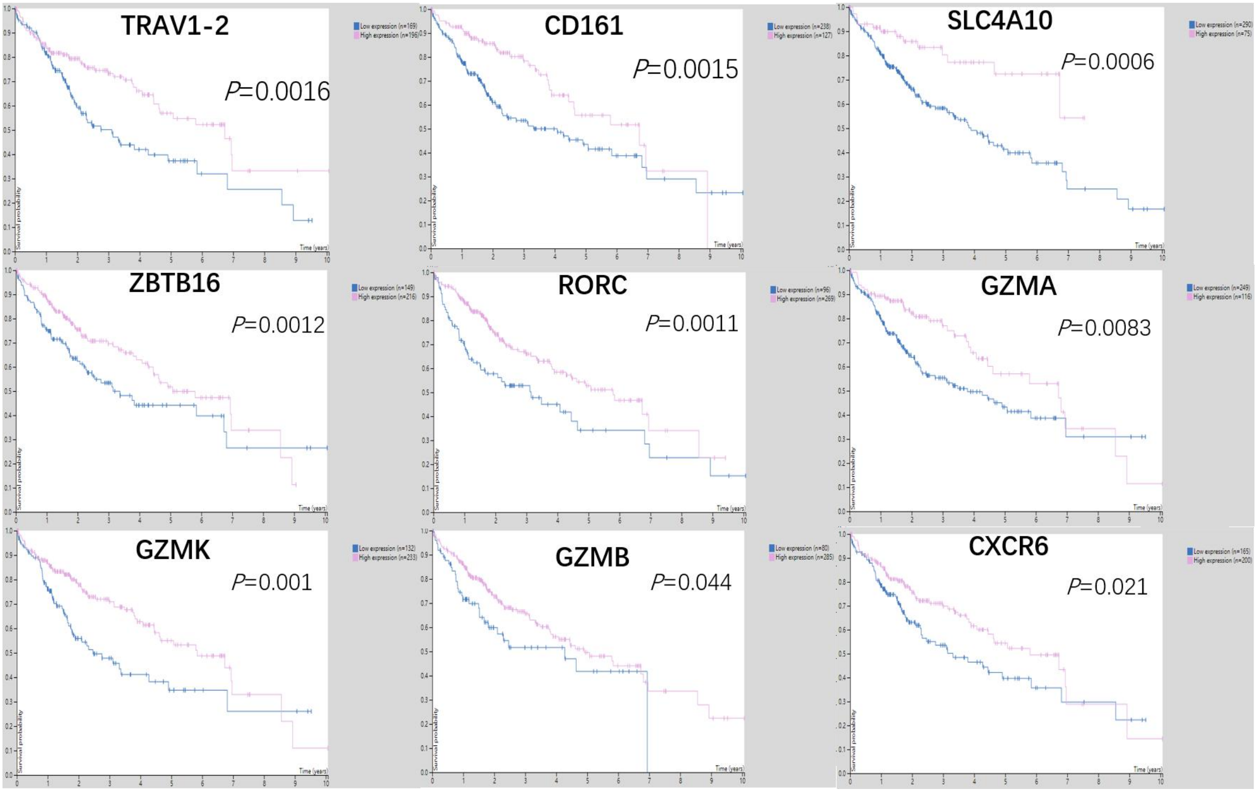

SI-Fig3. The association between marker genes of naïve B cells, MAIT cells, and NKT cells and the survival rate.

SI-FIGURE 4

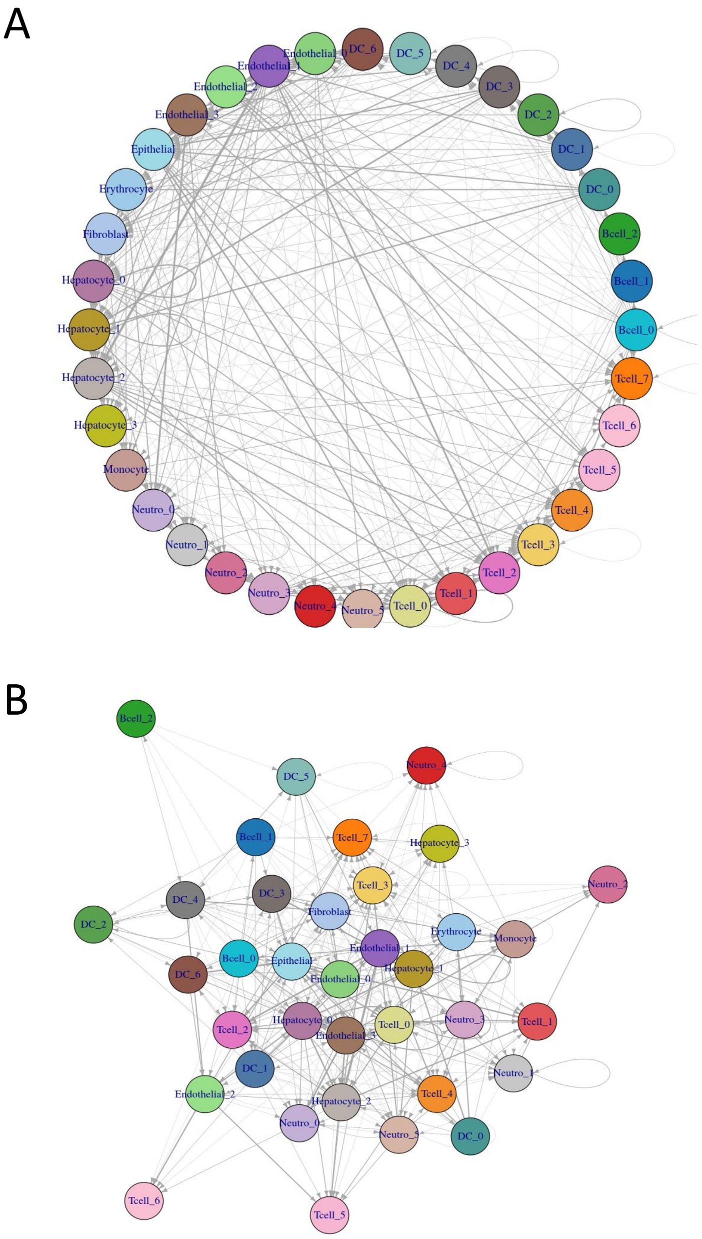

SI-Fig 4. Cell communication predicted by CellPhoneDB (a). The interaction between each cluster (b).

SI-FIGURE 5

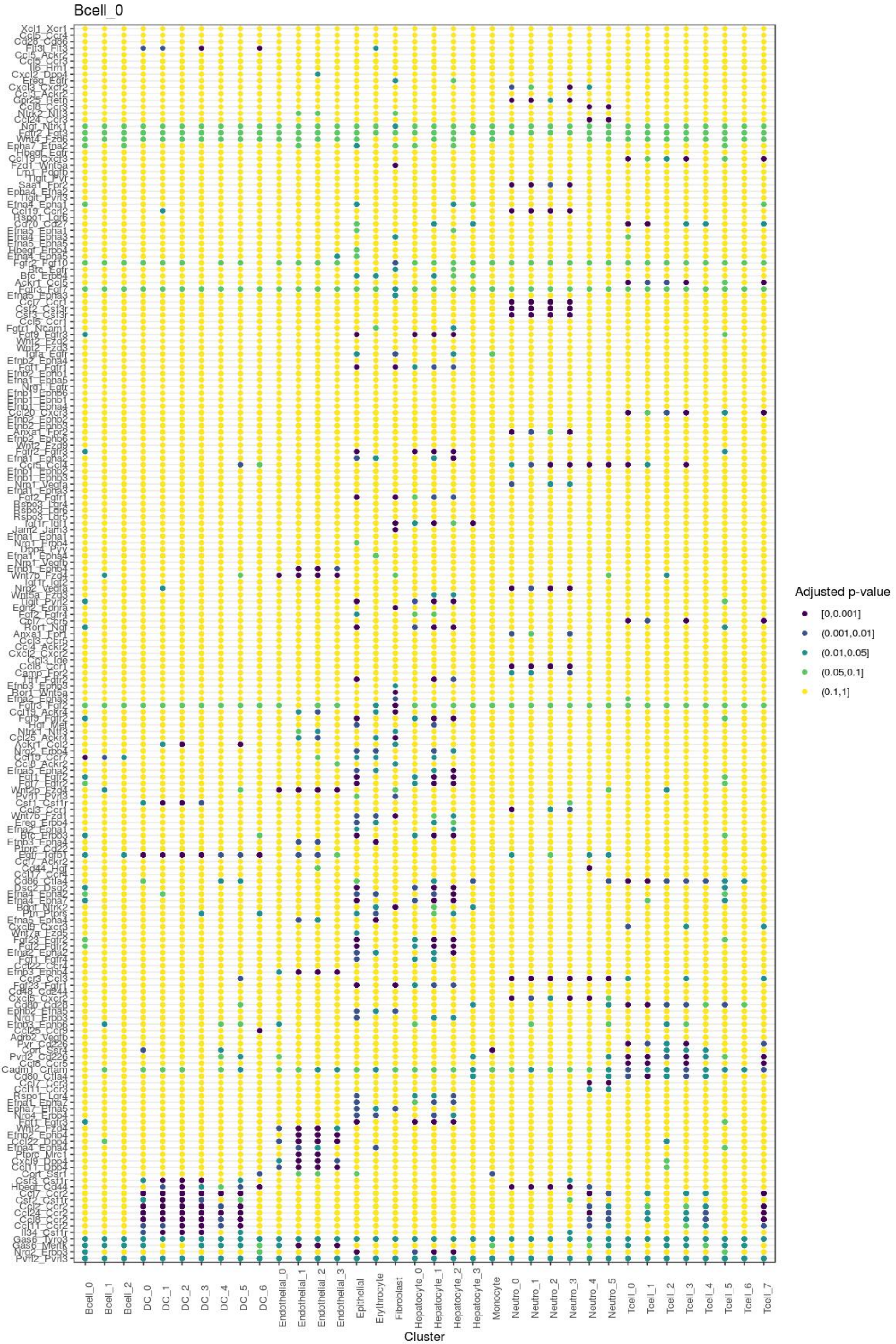

SI-Fig 5. Cell communication predicted by CellPhoneDB. Overview of selected ligand–receptor interactions; *P* values indicated by color (permutation test, see Methods).

Supplementary -Table 1 Single-cell transcriptomic characteristics of each sample

	**Pre-tumor**		**Cancer**	
	Mouse 1	Mouse 2	Mouse 3	
			Tumor	Adjacent
Number of cells	2124	2290	1572	1930
Mean reads/cell	102713	90609	124021	165148
Median Genes/cell	305	674	623	930

Supplementary -Table 2 Marker genes for T cell and B cell subset annotation

	Name	Description	Key markers	Notes
1	Tcell_0	T0-Exhausted CD8+T cell	Lag3, Ctla3, Pdcd1, Havcr2/Tim-3	Dominant in both adjacent and tumor tissue of the HCC late stage
2	Tcell_1	T1_ MAIT	Slc4a10, Zbtb16, Rorc	Composed of cells from adjacent and tumor tissue of the HCC late stage/ mucosal-associated invariant T cells
3	Tcell_2	T2-Cd38+T cell	Cd55, Cd38, Cd79, Cd74	Did not show significant different between each stage
4	Tcell_3	T3-naïve T cell	Lef1, Fcgr3, Tcf7	Early stage, did not show significant different between tumor and adjacent
5	Tcell_4	T4-NKT	Gzma, Gzmk, Gzmb, Cxcr6	predominantly composed of cells from early stage
6	Tcell_5	T5-Irf8+T	Irf8, Cd74	predominantly composed of cells from adjacent and tumor tissue of the HCC late stage
7	Tcell_6	T6_Cd4+Treg cell	Cd4+, Cd25, Foxp3	predominantly composed of cells from early stage
8	Tcell_7	T7_Effctor T	Cxccr1 Fcgr3a	T cells with effector functions

	Name	Description	Key markers	Notes
	**B cell_pan marker**	B cell	Cd79a, Cd79b, Cd19	
**1**	**B cell_0**	B0_Naïve B cell	Cd83, Irs2	increase in the proportion from the early to late stage tumorigenesis
**2**	**B cell_1**	B1_Plasmablast	Cd27, Cd38, Slamf7	dramatically decrease in the late stage
**3**	**B cell_2**	B2_Memory B	Cd27, Cd38, Cd40	dramatically decrease in the late stage

Supplementary -Table 3 Changes of subset proportions from early to late stages

**Subsets**	**Transition trends from early to late stage**
T0- Exhausted CD8+T cell	↑
T1_ MAIT	↑
T2-Cd38+T cell	--
T3-naïve T cell	↓
T4-NKT	↓
T5-Irf8+T	↑
T6_Cd4+T cell	↓
T7_Effector T	↓
B0_ Naïve B cell	↑
B1_ Plasmablast	↓
B2_ Memory B	↓
DC0	↓
DC1	--
DC2	↑
DC3	--
DC4	--
DC5	↓
DC6	↑
Endothelial0	--
Endothelial1	↑
Endothelial2	--
Endothelial3	↓
Neutrophil0	↓
Neutrophil1	--

Neutrophil2	--
Neutrophil3	--
Neutrophil4	↑
Neutrophil5	↑
Neutrophil6	↓
Hepatocyte0	↓
Hepatocyte1	↓
Hepatocyte2	↓
Hepatocyte3	↑

Supplementary -Table 4 Patterns of cell-cell communication

Subsets	Originating	Terminal	Self-regulation
T0		√	
T1		√	
T2		√	
T3		√	
T4		√	
T5		√	
T6		√	
T7		√	
B0	√		√
B1	√		
B2	√		
Hepa0			√
Hepa1			√
Hepa2		√	
Hepa3		√	

Supplementary -Table 5 Clinical information of TCGA HCC patient cohort

Sample	Description
TCGA-2Y-A9GS-01A	58 years, male, white, dead, 724 days
TCGA-2Y-A9GT-01A	51 years, male, white, stage:i, dead, 1624 days
TCGA-2Y-A9GU-01A	55 years, female, white, stage:i, alive, 1939 days
TCGA-2Y-A9GV-01A	54 years, female, white, stage:i, dead, 2532 days
TCGA-2Y-A9GW-01A	64 years, male, white, stage:i, dead, 1271 days
TCGA-2Y-A9GX-01A	68 years, male, white, stage:i, alive, 2442 days
TCGA-2Y-A9GY-01A	64 years, female, white, stage:ii, dead, 757 days
TCGA-2Y-A9GZ-01A	82 years, female, white, stage:ii, dead, 848 days
TCGA-2Y-A9H0-01A	49 years, male, white, stage:iiia, alive, 3675 days
TCGA-2Y-A9H1-01A	58 years, male, white, stage:i, dead, 1229 days
TCGA-2Y-A9H2-01A	64 years, female, white, stage:i, alive, 1731 days
TCGA-2Y-A9H3-01A	45 years, male, white, stage:ii, alive, 1516 days
TCGA-2Y-A9H4-01A	68 years, male, black or african american, stage:i, alive, 1452 days
TCGA-2Y-A9H5-01A	59 years, female, white, stage:i, dead, 555 days
TCGA-2Y-A9H6-01A	68 years, female, white, stage:i, alive, 357 days
TCGA-2Y-A9H7-01A	81 years, female, white, stage:i, alive, 1168 days
TCGA-2Y-A9H8-01A	85 years, female, white, dead, 633 days
TCGA-2Y-A9H9-01A	70 years, male, white, stage:i, alive, 697 days
TCGA-2Y-A9HA-01A	70 years, male, white, stage:ii, dead, 36 days
TCGA-2Y-A9HB-01A	66 years, male, stage:i, alive, 260 days
TCGA-3K-AAZ8-01A	65 years, male, black or african american, stage:iiib, alive, 396 days
TCGA-4R-AA8I-01A	66 years, male, white, stage:ii, dead, 262 days
TCGA-5C-A9VG-01A	58 years, male, white, stage:ii, alive, 328 days
TCGA-5C-A9VH-01A	70 years, male, white, stage:i, alive, 322 days
TCGA-5C-AAPD-01A	61 years, male, asian, stage:ii, alive, 20 days
TCGA-5R-AA1C-01A	57 years, male, white, stage:ii, alive, 520 days

TCGA-5R-AA1D-01A	17 years, female, white, stage:iiia, alive, 449 days
TCGA-5R-AAAM-01A	65 years, female, white, stage:ii, dead, 46 days
TCGA-BC-4072-01B	74 years, female, white, stage:iiia, dead, 1490 days
TCGA-BC-4073-01B	73 years, male, white, stage:iiia, alive, 849 days
TCGA-BC-A10Q-01A	72 years, female, white, dead, 1135 days
TCGA-BC-A10R-01A	66 years, female, white, dead, 308 days
TCGA-BC-A10S-01A	81 years, male, white, dead, 1423 days
TCGA-BC-A10T-01A	76 years, male, white, dead, 837 days
TCGA-BC-A10U-01A	69 years, male, white, dead, 837 days
TCGA-BC-A10W-01A	50 years, male, asian, dead, 91 days
TCGA-BC-A10X-01A	52 years, female, white, stage:iiia, dead, 770 days
TCGA-BC-A10Y-01A	76 years, male, white, dead, 711 days
TCGA-BC-A10Z-01A	62 years, female, white, stage:i, dead, 34 days
TCGA-BC-A110-01A	51 years, female, black or african american, dead, 2116 days
TCGA-BC-A112-01A	80 years, male, white, dead, 153 days
TCGA-BC-A216-01A	62 years, female, white, stage:iiia, alive, 1351 days
TCGA-BC-A217-01A	75 years, female, white, stage:ii, dead, 1397 days
TCGA-BC-A3KF-01A	66 years, female, white, stage:i, alive, 8 days
TCGA-BC-A3KG-01A	68 years, female, white, stage:ii, alive, 680 days
TCGA-BC-A5W4-01A	69 years, male, white, stage:iiia, dead, 547 days
TCGA-BC-A69H-01A	64 years, male, white, stage:ii, alive, 444 days
TCGA-BC-A69I-01A	69 years, male, white, stage:i, alive, 387 days
TCGA-BC-A8YO-01A	66 years, female, white, stage:iiic, alive, 562 days
TCGA-BD-A2L6-01A	69 years, male, white, alive, 1363 days
TCGA-BD-A3EP-01A	76 years, female, black or african american, stage:i, alive, 409 days
TCGA-BD-A3ER-01A	62 years, male, white, stage:ii, alive, 1115 days
TCGA-BW-A5NO-01A	50 years, male, black or african american, stage:iiia, alive, 20 days
TCGA-CC-5258-01A	48 years, male, asian, stage:ii, dead, 129 days
TCGA-CC-5259-01A	60 years, female, asian, stage:iiic, alive, 250 days

TCGA-CC-5260-01A	61 years, female, asian, stage:iiic, dead, 87 days
TCGA-CC-5261-01A	44 years, male, asian, stage:ii, dead, 97 days
TCGA-CC-5262-01A	67 years, male, asian, stage:iiic, dead, 103 days
TCGA-CC-5263-01A	35 years, male, asian, stage:iiia, dead, 129 days
TCGA-CC-5264-01A	71 years, male, asian, stage:iiia, dead, 102 days
TCGA-CC-A123-01A	24 years, female, asian, stage:iiia, alive, 219 days
TCGA-CC-A1HT-01A	50 years, male, asian, stage:iiia, dead, 101 days
TCGA-CC-A3M9-01A	45 years, male, asian, stage:iiia, dead, 300 days
TCGA-CC-A3MA-01A	61 years, male, asian, stage:iiia, dead, 303 days
TCGA-CC-A3MB-01A	36 years, male, asian, stage:iiia, dead, 315 days
TCGA-CC-A3MC-01A	54 years, male, asian, stage:iiia, alive, 363 days
TCGA-CC-A5UC-01A	63 years, male, asian, stage:iiia, dead, 347 days
TCGA-CC-A5UD-01A	45 years, male, asian, stage:iiia, dead, 304 days
TCGA-CC-A5UE-01A	48 years, male, asian, stage:iiib, dead, 272 days
TCGA-CC-A7IE-01A	57 years, male, asian, stage:iiia, dead, 217 days
TCGA-CC-A7IF-01A	59 years, male, asian, stage:iiia, dead, 649 days
TCGA-CC-A7IG-01A	47 years, male, asian, stage:ii, dead, 299 days
TCGA-CC-A7IH-01A	58 years, male, asian, stage:iiia, alive, 365 days
TCGA-CC-A7II-01A	55 years, male, asian, stage:iiia, alive, 399 days
TCGA-CC-A7IJ-01A	56 years, male, asian, stage:ii, alive, 382 days
TCGA-CC-A7IK-01A	59 years, male, asian, stage:iiia, dead, 262 days
TCGA-CC-A7IL-01A	61 years, male, asian, stage:iiia, dead, 278 days
TCGA-CC-A8HS-01A	18 years, male, asian, stage:iiic, dead, 300 days
TCGA-CC-A8HT-01A	74 years, male, asian, stage:iiia, dead, 140 days
TCGA-CC-A8HU-01A	39 years, female, asian, stage:iiia, dead, 344 days
TCGA-CC-A8HV-01A	51 years, female, asian, stage:ii, dead, 279 days
TCGA-CC-A9FS-01A	55 years, male, asian, stage:ii, alive, 211 days
TCGA-CC-A9FW-01A	68 years, male, asian, stage:iiia, alive, 248 days
TCGA-DD-A113-01A	55 years, female, white, stage:ii, alive, 2425 days

TCGA-DD-A114-01A	42 years, male, black or african american, stage:ii, dead, 1149 days
TCGA-DD-A115-01A	53 years, male, white, stage:iiia, dead, 2542 days
TCGA-DD-A116-01A	68 years, male, asian, stage:iiia, dead, 1622 days
TCGA-DD-A118-01A	77 years, female, white, stage:ii, alive, 3437 days
TCGA-DD-A119-01A	40 years, male, asian, stage:iv, dead, 223 days
TCGA-DD-A11A-01A	67 years, male, black or african american, stage:i, alive, 79 days
TCGA-DD-A11B-01A	73 years, male, white, stage:i, dead, 14 days
TCGA-DD-A11C-01A	69 years, male, white, stage:i, alive, 662 days
TCGA-DD-A11D-01A	57 years, female, white, stage:i, dead, 1560 days
TCGA-DD-A1EA-01A	68 years, male, asian, stage:ii, alive, 2415 days
TCGA-DD-A1EB-01A	72 years, female, stage:i, alive, 2017 days
TCGA-DD-A1EC-01A	20 years, female, white, stage:i, alive, 602 days
TCGA-DD-A1ED-01A	68 years, male, white, stage:i, alive, 2301 days
TCGA-DD-A1EE-01A	73 years, male, white, stage:iiia, dead, 349 days
TCGA-DD-A1EF-01A	57 years, female, white, stage:i, dead, 394 days
TCGA-DD-A1EG-01A	77 years, male, white, stage:i, dead, 1372 days
TCGA-DD-A1EH-01A	23 years, male, white, stage:iii, alive, 1495 days
TCGA-DD-A1EI-01A	46 years, male, asian, stage:i, alive, 183 days
TCGA-DD-A1EJ-01A	71 years, female, white, stage:iiic, dead, 1005 days
TCGA-DD-A1EK-01A	64 years, female, white, stage:ivb, dead, 558 days
TCGA-DD-A1EL-01A	23 years, male, black or african american, stage:ii, dead, 415 days
TCGA-DD-A39V-01A	77 years, male, white, stage:ii, dead, 643 days
TCGA-DD-A39W-01A	29 years, female, white, stage:iii, dead, 827 days
TCGA-DD-A39X-01A	78 years, female, white, stage:i, dead, 1694 days
TCGA-DD-A39Y-01A	67 years, male, asian, stage:i, dead, 171 days
TCGA-DD-A39Z-01A	43 years, female, stage:ii, dead, 601 days
TCGA-DD-A3A1-01A	65 years, male, stage:iiia, dead, 233 days
TCGA-DD-A3A2-01A	76 years, female, white, stage:i, dead, 2131 days
TCGA-DD-A3A3-01A	45 years, male, asian, stage:i, dead, 535 days

TCGA-DD-A3A4-01A	37 years, male, white, stage:iiia, dead, 612 days
TCGA-DD-A3A5-01A	66 years, female, white, stage:iii, dead, 3125 days
TCGA-DD-A3A6-01A	72 years, female, white, stage:ii, dead, 3258 days
TCGA-DD-A3A7-01A	67 years, male, stage:iiib, dead, 419 days
TCGA-DD-A3A8-01A	75 years, male, white, stage:ii, dead, 11 days
TCGA-DD-A3A9-01A	64 years, female, white, stage:ivb, dead, 931 days
TCGA-DD-A4NA-01A	67 years, female, white, stage:iiic, alive, 1008 days
TCGA-DD-A4NB-01A	25 years, male, white, stage:i, alive, 989 days
TCGA-DD-A4ND-01A	56 years, female, white, stage:i, alive, 2746 days
TCGA-DD-A4NE-01A	75 years, female, white, stage:iiia, dead, 660 days
TCGA-DD-A4NF-01A	72 years, male, white, stage:i, alive, 942 days
TCGA-DD-A4NG-01A	77 years, male, white, stage:iiia, dead, 802 days
TCGA-DD-A4NH-01A	65 years, female, white, stage:iiib, alive, 917 days
TCGA-DD-A4NI-01A	67 years, male, white, stage:ii, alive, 816 days
TCGA-DD-A4NJ-01A	54 years, female, white, stage:ii, alive, 928 days
TCGA-DD-A4NK-01A	80 years, female, white, stage:iiia, dead, 1210 days
TCGA-DD-A4NL-01A	46 years, male, white, stage:i, alive, 1711 days
TCGA-DD-A4NN-01A	56 years, female, white, stage:i, dead, 899 days
TCGA-DD-A4NO-01A	66 years, male, white, stage:i, alive, 2245 days
TCGA-DD-A4NP-01A	32 years, male, white, stage:i, alive, 3308 days
TCGA-DD-A4NQ-01A	60 years, male, white, stage:ii, dead, 373 days
TCGA-DD-A4NR-01A	85 years, female, white, stage:i, dead, 9 days
TCGA-DD-A4NS-01A	62 years, female, white, stage:i, dead, 2456 days
TCGA-DD-A4NV-01A	61 years, male, white, stage:iiia, alive, 2398 days
TCGA-DD-A73A-01A	71 years, male, white, stage:i, alive, 728 days
TCGA-DD-A73B-01A	72 years, female, white, stage:i, dead, 283 days
TCGA-DD-A73C-01A	65 years, female, white, stage:iiia, alive, 701 days
TCGA-DD-A73D-01A	68 years, female, white, stage:ii, alive, 693 days
TCGA-DD-A73E-01A	66 years, male, white, stage:i, alive, 44 days

TCGA-DD-A73F-01A	77 years, female, white, stage:i, alive, 1085 days
TCGA-DD-A73G-01A	73 years, female, white, stage:i, alive, 3478 days
TCGA-DD-AA3A-01A	81 years, female, white, stage:i, dead, 410 days
TCGA-DD-AAC8-01A	72 years, male, asian, stage:i, dead, 16 days
TCGA-DD-AAC9-01A	51 years, male, asian, stage:i, alive, 347 days
TCGA-DD-AACA-01A	65 years, male, asian, stage:i, alive, 2301 days
TCGA-DD-AACB-01A	74 years, female, asian, stage:i, alive, 2324 days
TCGA-DD-AACC-01A	61 years, male, asian, stage:i, dead, 1685 days
TCGA-DD-AACD-01A	48 years, male, asian, stage:i, dead, 381 days
TCGA-DD-AACE-01A	62 years, male, asian, stage:i, alive, 2184 days
TCGA-DD-AACF-01A	68 years, male, asian, stage:i, dead, 365 days
TCGA-DD-AACG-01A	52 years, male, asian, stage:ii, dead, 469 days
TCGA-DD-AACH-01A	69 years, male, asian, stage:ii, dead, 195 days
TCGA-DD-AACI-01A	69 years, male, asian, stage:ii, alive, 1618 days
TCGA-DD-AACJ-01A	75 years, male, asian, stage:ii, alive, 2102 days
TCGA-DD-AACK-01A	70 years, male, asian, stage:i, alive, 9 days
TCGA-DD-AACL-01A	66 years, female, asian, stage:i, dead, 107 days
TCGA-DD-AACN-01A	32 years, male, asian, stage:i, alive, 1302 days
TCGA-DD-AACO-01A	40 years, male, asian, stage:i, alive, 1876 days
TCGA-DD-AACP-01A	65 years, male, asian, stage:i, alive, 415 days
TCGA-DD-AACQ-01A	50 years, male, asian, stage:ii, dead, 432 days
TCGA-DD-AACS-01A	39 years, male, asian, stage:i, alive, 1804 days
TCGA-DD-AACT-01A	69 years, female, asian, stage:i, alive, 1562 days
TCGA-DD-AACU-01A	59 years, male, asian, stage:i, alive, 1567 days
TCGA-DD-AACV-01A	53 years, male, asian, stage:i, alive, 1531 days
TCGA-DD-AACW-01A	43 years, male, asian, stage:i, alive, 1424 days
TCGA-DD-AACX-01A	66 years, male, asian, stage:ii, alive, 170 days
TCGA-DD-AACY-01A	61 years, male, asian, stage:i, alive, 1450 days
TCGA-DD-AACZ-01A	63 years, female, asian, stage:i, dead, 171 days

TCGA-DD-AAD0-01A	73 years, female, asian, stage:i, alive, 137 days
TCGA-DD-AAD1-01A	51 years, female, asian, stage:i, alive, 564 days
TCGA-DD-AAD2-01A	66 years, male, asian, stage:i, alive, 658 days
TCGA-DD-AAD3-01A	43 years, male, asian, stage:i, alive, 1295 days
TCGA-DD-AAD5-01A	54 years, male, asian, stage:i, alive, 1345 days
TCGA-DD-AAD6-01A	66 years, male, asian, stage:iiia, alive, 672 days
TCGA-DD-AAD8-01A	73 years, female, asian, stage:i, alive, 1219 days
TCGA-DD-AADA-01A	66 years, female, asian, stage:i, alive, 1233 days
TCGA-DD-AADB-01A	51 years, male, asian, stage:i, alive, 1242 days
TCGA-DD-AADC-01A	53 years, male, asian, stage:i, dead, 425 days
TCGA-DD-AADD-01A	51 years, male, asian, stage:i, alive, 1231 days
TCGA-DD-AADF-01A	64 years, female, asian, stage:i, dead, 115 days
TCGA-DD-AADG-01A	70 years, male, asian, stage:iiia, alive, 1145 days
TCGA-DD-AADI-01A	43 years, female, asian, stage:i, alive, 1085 days
TCGA-DD-AADJ-01A	70 years, female, asian, stage:i, alive, 1066 days
TCGA-DD-AADK-01A	68 years, female, asian, stage:ii, alive, 1049 days
TCGA-DD-AADL-01A	58 years, male, asian, stage:i, alive, 636 days
TCGA-DD-AADM-01A	58 years, male, asian, stage:ii, dead, 12 days
TCGA-DD-AADN-01A	59 years, male, asian, stage:i, alive, 898 days
TCGA-DD-AADO-01A	55 years, male, asian, stage:i, alive, 453 days
TCGA-DD-AADP-01A	45 years, male, asian, stage:i, alive, 458 days
TCGA-DD-AADQ-01A	59 years, male, asian, stage:ii, alive, 436 days
TCGA-DD-AADR-01A	58 years, male, asian, stage:i, alive, 2028 days
TCGA-DD-AADS-01A	63 years, male, asian, stage:i, alive, 474 days
TCGA-DD-AADU-01A	60 years, male, asian, stage:ii, alive, 554 days
TCGA-DD-AADV-01A	50 years, male, asian, stage:i, alive, 574 days
TCGA-DD-AADW-01A	48 years, male, asian, stage:i, alive, 587 days
TCGA-DD-AADY-01A	55 years, female, asian, stage:i, alive, 555 days
TCGA-DD-AAE0-01A	45 years, female, asian, stage:iiia, alive, 555 days

TCGA-DD-AAE1-01A	52 years, male, asian, stage:i, alive, 552 days
TCGA-DD-AAE2-01A	51 years, male, asian, stage:i, alive, 638 days
TCGA-DD-AAE3-01A	50 years, male, asian, stage:i, alive, 566 days
TCGA-DD-AAE4-01A	49 years, female, asian, stage:i, alive, 608 days
TCGA-DD-AAE6-01A	59 years, female, asian, stage:i, alive, 141 days
TCGA-DD-AAE7-01A	72 years, male, asian, stage:i, alive, 644 days
TCGA-DD-AAE9-01A	69 years, male, asian, stage:i, alive, 722 days
TCGA-DD-AAEA-01A	65 years, male, asian, stage:i, alive, 575 days
TCGA-DD-AAEB-01A	60 years, male, asian, stage:i, alive, 478 days
TCGA-DD-AAED-01A	51 years, male, asian, stage:i, alive, 763 days
TCGA-DD-AAEE-01A	55 years, male, asian, stage:i, alive, 810 days
TCGA-DD-AAEG-01A	59 years, female, asian, stage:i, alive, 719 days
TCGA-DD-AAEH-01A	73 years, male, asian, stage:i, alive, 784 days
TCGA-DD-AAEI-01A	72 years, male, asian, stage:i, alive, 1531 days
TCGA-DD-AAEK-01A	51 years, male, asian, stage:ii, alive, 1067 days
TCGA-DD-AAVP-01A	48 years, male, asian, stage:i, alive, 2752 days
TCGA-DD-AAVQ-01A	38 years, male, asian, stage:i, alive, 2728 days
TCGA-DD-AAVR-01A	44 years, male, asian, stage:i, alive, 2513 days
TCGA-DD-AAVS-01A	56 years, male, asian, stage:i, alive, 1823 days
TCGA-DD-AAVU-01A	46 years, male, asian, stage:ii, alive, 2202 days
TCGA-DD-AAVV-01A	56 years, male, asian, stage:ii, alive, 2455 days
TCGA-DD-AAVW-01A	35 years, male, asian, stage:i, alive, 2317 days
TCGA-DD-AAVX-01A	38 years, male, asian, stage:ii, alive, 1570 days
TCGA-DD-AAVY-01A	56 years, male, asian, stage:iiia, alive, 1970 days
TCGA-DD-AAVZ-01A	38 years, male, asian, stage:i, alive, 1900 days
TCGA-DD-AAW0-01A	54 years, male, asian, stage:i, alive, 2015 days
TCGA-DD-AAW1-01A	55 years, male, asian, stage:iiia, alive, 1989 days
TCGA-DD-AAW2-01A	69 years, male, asian, stage:i, alive, 1855 days
TCGA-DD-AAW3-01A	69 years, male, asian, stage:i, alive, 1633 days

TCGA-ED-A459-01A	47 years, male, asian, stage:ii, alive, 910 days
TCGA-ED-A4XI-01A	58 years, male, asian, stage:ii, alive, 819 days
TCGA-ED-A5KG-01A	60 years, female, asian, stage:ii, alive, 854 days
TCGA-ED-A627-01A	74 years, male, white, stage:i, alive, 423 days
TCGA-ED-A66X-01A	35 years, male, asian, stage:iiia, alive, 406 days
TCGA-ED-A66Y-01A	51 years, female, asian, stage:iiia, dead, 296 days
TCGA-ED-A7PX-01A	48 years, female, asian, stage:ii, alive, 6 days
TCGA-ED-A7PY-01A	20 years, female, asian, stage:ii, alive, 390 days
TCGA-ED-A7PZ-01A	61 years, male, asian, stage:ii, alive, 6 days
TCGA-ED-A7XO-01A	29 years, male, asian, stage:iiia, alive, 427 days
TCGA-ED-A7XP-01A	53 years, female, asian, stage:ii, alive, 400 days
TCGA-ED-A82E-01A	60 years, female, asian, stage:iiia, alive, 408 days
TCGA-ED-A8O5-01A	59 years, female, asian, stage:iiia, alive, 406 days
TCGA-ED-A8O6-01A	50 years, female, asian, stage:iiia, dead, 56 days
TCGA-ED-A97K-01A	54 years, male, asian, stage:iiia, alive, 6 days
TCGA-EP-A12J-01A	63 years, male, black or african american, stage:i, alive, 570 days
TCGA-EP-A26S-01A	70 years, male, white, stage:i, alive, 608 days
TCGA-EP-A2KA-01A	52 years, female, white, stage:iiia, dead, 627 days
TCGA-EP-A2KB-01A	46 years, female, white, stage:i, dead, 596 days
TCGA-EP-A2KC-01A	62 years, male, black or african american, stage:i, dead, 19 days
TCGA-EP-A3JL-01A	76 years, male, white, stage:i, alive, 303 days
TCGA-EP-A3RK-01A	73 years, male, white, stage:iiia, alive, 363 days
TCGA-ES-A2HS-01A	80 years, male, white, stage:i, dead, 688 days
TCGA-ES-A2HT-01A	54 years, male, black or african american, stage:i, dead, 438 days
TCGA-FV-A23B-01A	70 years, female, white, stage:ii, dead, 1852 days
TCGA-FV-A2QQ-01A	80 years, male, white, stage:i, alive, 729 days
TCGA-FV-A2QR-01A	male, white, stage:i, dead, 581 days
TCGA-FV-A3I0-01A	76 years, female, white, stage:ii, alive, 848 days
TCGA-FV-A3I1-01A	female, white, stage:ii, dead, 247 days

TCGA-FV-A3R2-01A	75 years, male, white, stage:i, dead, 194 days
TCGA-FV-A3R3-01A	38 years, female, white, stage:i, dead, 366 days
TCGA-FV-A495-01A	51 years, female, white, stage:ii, alive, 1 days
TCGA-FV-A496-01A	84 years, female, white, stage:i, alive, 10 days
TCGA-FV-A4ZP-01A	78 years, male, white, stage:iiia, dead, 2486 days
TCGA-FV-A4ZQ-01A	52 years, male, white, stage:i, alive, 12 days
TCGA-G3-A25S-01A	64 years, male, white, stage:i, dead, 416 days
TCGA-G3-A25T-01A	45 years, female, white, stage:iiia, alive, 1553 days
TCGA-G3-A25U-01A	63 years, female, asian, stage:i, alive, 1636 days
TCGA-G3-A25V-01A	68 years, male, white, stage:i, alive, 860 days
TCGA-G3-A25X-01A	73 years, male, asian, stage:ii, alive, 1779 days
TCGA-G3-A25Y-01A	52 years, female, asian, stage:i, dead, 452 days
TCGA-G3-A25Z-01A	58 years, male, asian, stage:i, alive, 655 days
TCGA-G3-A3CG-01A	80 years, male, white, stage:i, alive, 673 days
TCGA-G3-A3CH-01A	53 years, male, asian, stage:iiia, alive, 780 days
TCGA-G3-A3CI-01A	71 years, male, white, stage:i, alive, 180 days
TCGA-G3-A3CJ-01A	52 years, male, american indian or alaska native, stage:ii, alive, 594 days
TCGA-G3-A3CK-01A	61 years, male, asian, stage:i, alive, 585 days
TCGA-G3-A5SI-01A	44 years, male, asian, stage:ii, dead, 768 days
TCGA-G3-A5SJ-01A	59 years, male, white, stage:i, alive, 698 days
TCGA-G3-A5SK-01A	58 years, male, white, stage:i, alive, 744 days
TCGA-G3-A5SL-01A	70 years, male, white, stage:ii, alive, 621 days
TCGA-G3-A5SM-01A	58 years, male, white, stage:ii, alive, 520 days
TCGA-G3-A6UC-01A	65 years, male, white, stage:iiib, alive, 671 days
TCGA-G3-A7M5-01A	76 years, male, asian, stage:i, alive, 447 days
TCGA-G3-A7M6-01A	60 years, female, white, stage:i, alive, 632 days
TCGA-G3-A7M7-01A	65 years, male, white, stage:i, alive, 361 days
TCGA-G3-A7M8-01A	31 years, male, asian, stage:i, alive, 430 days
TCGA-G3-A7M9-01A	70 years, male, white, stage:iiib, dead, 56 days

TCGA-G3-AAUZ-01A	48 years, male, stage:i, alive, 480 days
TCGA-G3-AAV0-01A	58 years, male, asian, stage:i, alive, 476 days
TCGA-G3-AAV1-01A	51 years, male, asian, stage:iiic, dead, 359 days
TCGA-G3-AAV2-01A	50 years, male, white, stage:i, alive, 372 days
TCGA-G3-AAV3-01A	58 years, female, white, stage:ii, alive, 412 days
TCGA-G3-AAV4-01A	83 years, female, white, stage:i, dead, 27 days
TCGA-G3-AAV5-01A	67 years, male, white, stage:ii, alive, 354 days
TCGA-G3-AAV6-01A	53 years, female, white, stage:iiia, dead, 65 days
TCGA-G3-AAV7-01A	38 years, male, asian, stage:ii, alive, 361 days
TCGA-GJ-A3OU-01A	59 years, male, white, stage:i, alive, 879 days
TCGA-GJ-A6C0-01A	75 years, female, white, stage:ii, dead, 31 days
TCGA-GJ-A9DB-01A	68 years, male, white, stage:i, dead, 67 days
TCGA-HP-A5MZ-01A	62 years, male, stage:i, dead, 91 days
TCGA-HP-A5N0-01A	88 years, female, dead, 1147 days
TCGA-K7-A5RF-01A	64 years, male, white, stage:i, alive, 631 days
TCGA-K7-A5RG-01A	66 years, male, black or african american, stage:i, alive, 519 days
TCGA-K7-A6G5-01A	66 years, male, white, stage:i, alive, 512 days
TCGA-K7-AAU7-01A	61 years, male, white, stage:ii, alive, 359 days
TCGA-KR-A7K0-01A	65 years, male, white, stage:i, dead, 65 days
TCGA-KR-A7K2-01A	64 years, male, white, stage:i, alive, 829 days
TCGA-KR-A7K7-01A	61 years, female, white, stage:ii, alive, 951 days
TCGA-KR-A7K8-01A	57 years, male, stage:i, alive, 906 days
TCGA-LG-A6GG-01A	79 years, female, white, stage:ii, alive, 387 days
TCGA-LG-A9QC-01A	48 years, male, white, stage:i, alive, 425 days
TCGA-LG-A9QD-01A	68 years, male, white, stage:iiia, alive, 366 days
TCGA-MI-A75C-01A	64 years, male, white, stage:i, alive, 291 days
TCGA-MI-A75E-01A	61 years, male, white, stage:iiic, alive, 507 days
TCGA-MI-A75G-01A	63 years, male, white, stage:ii, alive, 698 days
TCGA-MI-A75H-01A	77 years, male, white, alive, 747 days

TCGA-MI-A75I-01A	61 years, male, black or african american, alive, 630 days
TCGA-MR-A520-01A	58 years, male, white, stage:i, alive, 229 days
TCGA-MR-A8JO-01A	34 years, male, white, stage:i, alive, 330 days
TCGA-NI-A4U2-01A	71 years, male, white, stage:iiia, dead, 1791 days
TCGA-NI-A8LF-01A	74 years, male, white, stage:i, alive, 799 days
TCGA-O8-A75V-01A	54 years, male, stage:i, alive, 538 days
TCGA-PD-A5DF-01A	58 years, female, white, stage:iiib, dead, 639 days
TCGA-QA-A7B7-01A	48 years, male, black or african american, stage:ii, alive, 94 days
TCGA-RC-A6M4-01A	74 years, female, white, stage:iiia, alive, 22 days
TCGA-RC-A6M5-01A	20 years, female, white, stage:iva, alive, 15 days
TCGA-RC-A6M6-01A	75 years, male, white, stage:ii, alive, 9 days
TCGA-RC-A7S9-01A	47 years, female, asian, stage:i, alive, 640 days
TCGA-RC-A7SB-01A	53 years, male, asian, stage:ii, alive, 588 days
TCGA-RC-A7SF-01A	66 years, male, asian, stage:i, alive, 579 days
TCGA-RC-A7SH-01A	42 years, male, asian, stage:ii, alive, 468 days
TCGA-RC-A7SK-01A	59 years, male, asian, stage:i, alive, 472 days
TCGA-RG-A7D4-01A	69 years, male, black or african american, stage:ii, alive, 1098 days
TCGA-T1-A6J8-01A	68 years, male, white, alive, 23 days
TCGA-UB-A7MA-01A	62 years, female, white, stage:ii, alive, 848 days
TCGA-UB-A7MB-01A	24 years, male, white, stage:ii, alive, 601 days
TCGA-UB-A7MC-01A	59 years, male, white, stage:iiia, alive, 500 days
TCGA-UB-A7MD-01A	67 years, male, black or african american, stage:i, dead, 52 days
TCGA-UB-A7ME-01A	51 years, male, asian, stage:i, alive, 486 days
TCGA-UB-A7MF-01A	57 years, male, white, stage:iiia, dead, 214 days
TCGA-UB-AA0U-01A	60 years, male, white, stage:ii, alive, 327 days
TCGA-UB-AA0V-01A	69 years, female, white, stage:i, alive, 314 days
TCGA-WJ-A86L-01A	68 years, female, white, stage:i, alive, 345 days
TCGA-WQ-A9G7-01A	female, white, alive, 30 days
TCGA-WQ-AB4B-01A	62 years, male, white, stage:ii, alive, 395 days

TCGA-WX-AA44-01A	64 years, female, white, stage:i, alive, 615 days
TCGA-WX-AA46-01A	62 years, male, white, stage:ii, alive, 756 days
TCGA-WX-AA47-01A	33 years, female, white, stage:iiia, dead, 556 days
TCGA-XR-A8TC-01A	43 years, female, white, stage:i, alive, 1339 days
TCGA-XR-A8TD-01A	49 years, female, white, stage:iiib, alive, 1030 days
TCGA-XR-A8TE-01A	16 years, male, white, stage:iiia, alive, 925 days
TCGA-XR-A8TF-01A	74 years, male, white, stage:i, dead, 693 days
TCGA-XR-A8TG-01A	59 years, male, white, stage:i, alive, 898 days
TCGA-YA-A8S7-01A	69 years, male, white, stage:iiia, dead, 412 days
TCGA-ZP-A9CV-01A	59 years, male, white, dead, 1088 days
TCGA-ZP-A9CY-01A	66 years, female, white, alive, 782 days
TCGA-ZP-A9CZ-01A	72 years, male, asian, alive, 706 days
TCGA-ZP-A9D0-01A	67 years, female, black or african american, alive, 1091 days
TCGA-ZP-A9D1-01A	56 years, female, white, alive, 21 days
TCGA-ZP-A9D2-01A	51 years, male, white, dead, 765 days
TCGA-ZP-A9D4-01A	64 years, female, white, alive, 395 days
TCGA-ZS-A9CD-01A	73 years, male, white, stage:ii, dead, 1386 days
TCGA-ZS-A9CE-01A	79 years, female, white, stage:ii, alive, 1241 days
TCGA-ZS-A9CF-01A	64 years, male, white, stage:ii, alive, 2412 days
TCGA-ZS-A9CG-01A	55 years, male, white, stage:ii, alive, 341 days

Supplementary -Table 6 Clinical information of three HCC patients

**Patient ID**	**Age**	**Gender**	**HBV antigens detection**	**HCV antigens detection**	**AFP(μg/L)**	**CY211(ng/mL)**	**CEA(μg/L)**	**NSE(ng/mL)**	**CA125(U/mL)**	**CA153(U/mL)**	**CA199(U/mL)**	**CA724(U/mL)**
HS-2	52	M	(-)	(-)	17547	2.02	2.73	10.23	9.06	6.31	39.15	0.84
HS-4	77	F	(-)	(-)	1122	5.33	2.25	10.39	11.24	6.78	12.94	＜0.2
HS-7	64	M	(-)	(-)	4.82	2.37	2.74	7.94	38.47	6.31	20.11	2.2
